# A Review on Multi-Level Asymmetric Design for 2D Neuromorphic Devices

**DOI:** 10.1007/s40820-026-02214-8

**Published:** 2026-05-06

**Authors:** Yilin Sun, Yuandong Gao, Zimu Wang, Zerui Cai, Zhifang Liu, Jianlong Xu

**Affiliations:** 1 School of Microelectronics Science and Technology, Sun Yat-sen University, Zhuhai, 519000 Guangdong People’s Republic of China; 2 Institute of Atomic Manufacturing, International Institute for Interdisciplinary and Frontiers, Beihang University, Beijing, 100191 People’s Republic of China; 3 Laboratory of Multi-spectral Absorbing Materials and Structures, Ministry of Education, University of Electronic Science and Technology of China, UESTC, Chengdu, People’s Republic of China; 4 Institute of Functional Nano & Soft Materials (FUNSOM), Jiangsu Key Laboratory for Carbon-Based Functional Materials & Devices, Soochow University, Suzhou, 215123 Jiangsu People’s Republic of China

**Keywords:** Asymmetric design, Anisotropic crystal structure, Band alignment engineering, Contact engineering, Neuromorphic devices

## Abstract

Asymmetry has emerged as a critical design strategy for implementing essential neuromorphic functionalities, such as directional signal propagation, programmable plasticity, and bio-inspired dynamics. This principle involves deliberately breaking symmetry at various scales, which introduces unique physical phenomena including spontaneous built-in fields, anisotropic carrier transport, and memristive switching, which are foundational to synaptic and neuronal emulation. Despite growing research, a systematic synthesis of how asymmetry function across different design levels is lacking, hindering the development of guiding design principles. This review bridges this gap by systematically examining asymmetric engineering across three levels: materials, structures, and device. Benefiting from the inherent structural and electronic versatility of two-dimensional (2D) materials, their unique physical properties arising from such multi-level asymmetry for emulating and regulating synaptic plasticity are analyzed to demonstrate the resultant functional diversity and tunability of neuromorphic devices. Furthermore, existing challenges are discussed and a forward-looking perspective on integrating multiple asymmetries and extending the concept to circuit and system levels is provided. This work aims to establish a coherent design framework and provide a unique pathway for developing next-generation neuromorphic intelligent hardware based on 2D materials.

## Highlights

- This review highlights the pivotal role of asymmetry as a core design strategy for realizing key neuromorphic functionalities by breaking symmetry across scales to induce unique physical phenomena.
- It systematically synthesizes asymmetric engineering at materials, structures, and device levels, with a focus on 2D materials to elaborate how their multi-level asymmetry enables tunable synaptic plasticity emulation.
- It addresses existing research gaps, discusses current challenges, and provides a forward-looking perspective to establish a coherent design framework for next-generation 2D material-based neuromorphic hardware.

## Introduction

The burgeoning field of neuromorphic intelligence hardware aims to transcend the limitations of von Neumann architecture by emulating the brain-like parallel, event-driven, and ultra-efficient computational principles [1]. While conventional silicon-based circuits have demonstrated rudimentary neuromorphic functions, they often struggle to replicate the brain’s fundamental building blocks, neurons and synapses, with the requisite energy efficiency, dynamic plasticity, and integration density [2]. In this context, two-dimensional (2D) materials have emerged as a revolutionary platform, offering exceptional advantages such as atomic-scale thickness, gate-tunable electronic properties, and rich band-structure engineering possibilities [3]. These inherent attributes have inspired diverse device engineering strategies, such as the fabrication of memristive crossbars from switchable 2D layers [4], the construction of heterojunctions for synaptic function simulation [5], and the design of synaptic transistors with optimized weight storage and updating [6].

However, the performance of these neuromorphic devices is often hampered by critical challenges, such as high switching variability, excessive power consumption, and inadequate linearity in synaptic weight updates. These issues predominantly stem from the stochastic and isotropic nature of charge transport in conventional symmetric material systems, which lacks the directional control and built-in biases inherent to biological neural networks [7, 8]. Addressing these limitations necessitates a shift toward more sophisticated design paradigms that can introduce deterministic control over physical processes. A key dimension of this effort involves exploiting engineered asymmetry across the material, interface, and device hierarchies to precisely tailor charge transport and synaptic dynamics.

The introduction of asymmetry in 2D materials serves as a fundamental strategy to directly modulate the core physical mechanisms underlying neuromorphic behaviors. By deliberately tailoring structural, interfacial, or geometric asymmetries of 2D materials, it becomes possible to govern charge drift versus stochastic diffusion, or anisotropic ion transport [9]. This deterministic control is crucial for emulating the sophisticated dynamics of biological synapses and neurons, enabling devices that not only mimic but also potentially surpass biological efficiency in specific metrics [10]. For example, the in-plane anisotropic optoelectronic properties of ReSe_2_ allows for the realization of the angle-dependent synaptic plasticity at device level, where the efficacy of synaptic weight updates can be modulated by aligning the input signal with specific crystal directions, thereby reducing switching variability and introducing a new degree of freedom for neural network design [11]. Besides, the asymmetric contact geometry in the α-In_2_Se_3_-based transistor enables self-powered neuromorphic vision, where the built-in electric field generated by unequal electrode areas facilitates zero-bias photodetection, adaptive light response, and optical memory formation, integrating perception and processing within a single energy-efficient platform [12]. Ultimately, by leveraging asymmetry as a core design principle, neuromorphic devices are transformed from passive elements into active and reconfigurable systems, paving the way for a new generation of versatile, low-power, and high-fidelity brain-inspired hardware.

**Fig. 1:**
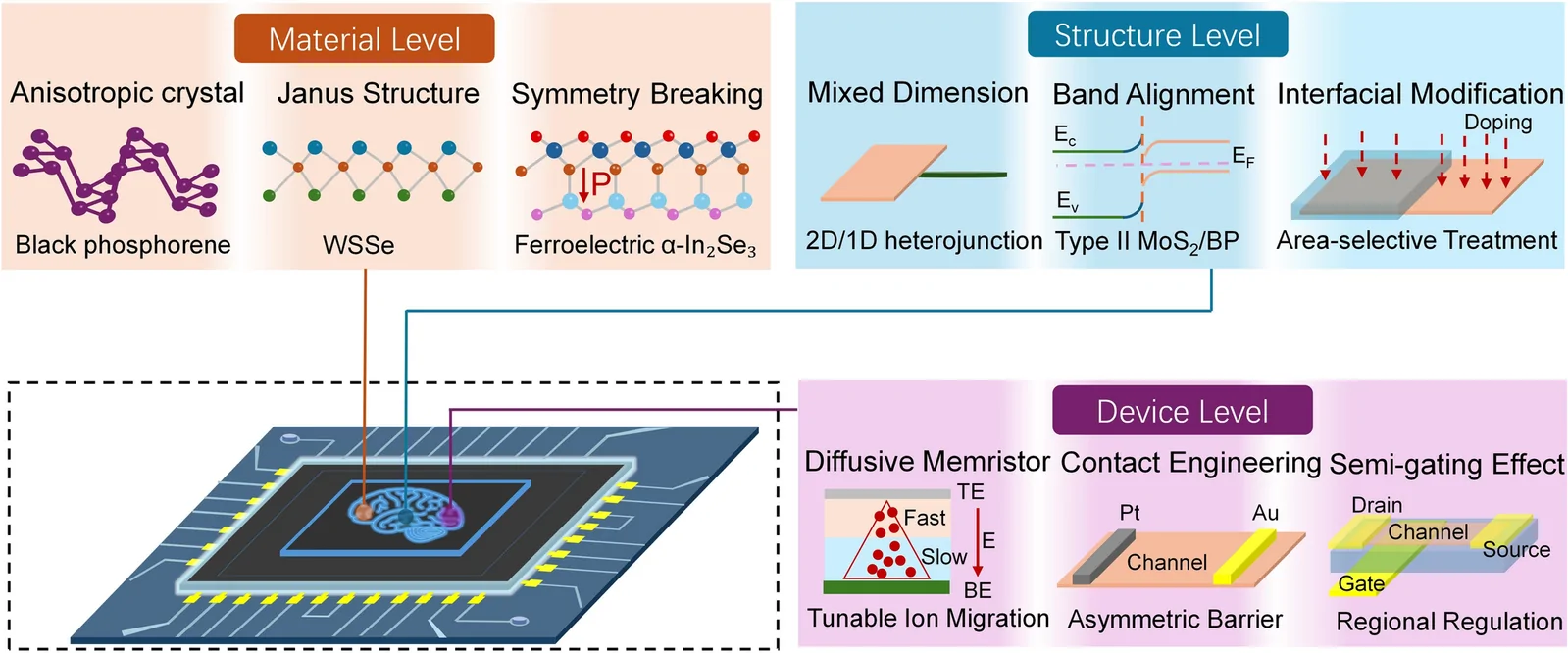
Overview of this review. Illustration of asymmetry engineering from multi-levels ranging from 2D materials, vdWs heterojunction, and device structure

This review introduces a hierarchical framework of multi-level engineering to systematically synthesize recent advancements in 2D neuromorphic devices, distinct from previous reviews by its focus on asymmetry as a universal design principle across three complementary scales: the material, heterostructure, and device architecture as illustrated schematically in Fig. 1. Here, we define “asymmetry” as the deliberate breaking of structural, compositional, or geometric symmetry within a material or device system to generate net vectorial properties such as directional charge transport or anisotropic optical responses that would otherwise be absent in perfectly symmetric configurations. This definition explicitly excludes random, stochastic variations arising from fabrication imperfections, focusing instead on engineered asymmetries that are intentionally introduced and spatially well defined, including crystallographic anisotropy in intrinsically low-symmetry 2D materials, compositional asymmetry in Janus structures and heterojunctions, interfacial asymmetry through selective functionalization, and geometric asymmetry in contact configuration or gate placement. What distinguishes this design philosophy from conventional approaches is its emphasis on harnessing asymmetry as a predictive and programmable degree of freedom, rather than treating it as an unavoidable deviation from an ideal symmetric baseline. Within this framework, we will elucidate how intrinsic symmetry breaking at the atomic level defines fundamental neuromorphic properties; how engineered interfacial asymmetry in van der Waals (vdWs) heterostructures creates novel synaptic functionalities; and how extrinsic geometric asymmetry in device design can be leveraged to enhance performance and enable new computing paradigms. By establishing this cross-scale perspective, this review aims not only to catalog progress but also to provide a unifying design strategy and a forward-looking outlook on the challenges and opportunities, thereby guiding the development of next-generation neuromorphic intelligence hardware.

## Material Level: Intrinsic Atomic Asymmetry

Intrinsic asymmetry at the atomic scale determines fundamental physical properties in 2D materials such as anisotropic charge transport [13], built-in electric field [14], and non-volatile switching characteristics [15]. These emergent properties provide a crucial foundation for neuromorphic functionalities by directly emulating biological processes such as directional signal propagation and dynamic synaptic weight modulation [16]. Prominent material-level strategies to harness this design principle include anisotropic crystal structures, Janus configurations, and symmetry-breaking-induced ferroelectric polarization in 2D materials. The following sections will elaborate on how these atomic-scale paradigms enable the hardware implementation of advanced neuromorphic intelligence devices.

### Anisotropic Crystal Structure

The anisotropic crystal structure in 2D materials is characterized by a low-symmetry lattice configuration where physical properties exhibit a strong directional dependence on the in-plane crystal direction. This intrinsic directionality originates from non-equivalent atomic bonding along different crystallographic axes, such as the distinct arrangements along the armchair versus zigzag directions in layered semiconductors. The structural anisotropy manifests in fundamental material properties including inherently directional charge transport [17], dichroic optical absorption [18], anisotropic thermal conductivity [19], and orientation-dependent mechanical response [20]. Table 1 summarizes the state-of-the-art anisotropic 2D materials with the highlighted physical properties, where the anisotropic ratio describes the difference of the corresponding characteristics obtained in different directions. These direction-dependent characteristics provide a robust physical basis for implementing vectorial functionalities in electronic devices. However, the selection of suitable anisotropic materials for neuromorphic applications requires careful consideration of how specific anisotropy types align with target computational functions. For instance, materials with high electrical anisotropic ratios may be preferred for directional charge transport in synaptic weight modulation, while those exhibiting strong optical anisotropy are better suited for polarization-sensitive vision systems. Mechanical anisotropy can be exploited in flexible or wearable neuromorphic platforms where direction-dependent strain responses are desired, while thermal anisotropy may be relevant for managing heat dissipation in high-density integrations. Thus, the choice of anisotropic material must align with the intended computational paradigm, considering which physical property offers the most direct and efficient pathway to implement the desired neuromorphic function.

**Table 1:** The summary of crystal structure and anisotropic physical properties of 2D materials

Component	Materials	Structure	Anisotropic ratio			
			Mobility	Photocurrent	Thermal conductivity	Young’s modulus
Simple substance	Black phosphorus (BP)		16 for [21]	8.7 at 1550 nm [22]	~ 0.5 for thick film ~ 0.67 for thin film [23]	~ 0.46 [24]
	Violet phosphorus (VP)		2.7 [25]	3.9 at 520 nm [25]	~ 1.33 for 200 nm thick [26]	–
	Te		1.42 [27]	4.6 at 1064 nm [28]	1.47 for 100 nm thick 2.67 for 15 nm thick [29]	~ 0.44 [30]
	Sb		–	2.4 at 496 nm [31]	/	~ 0.29 [32]
Compound	PdSe_2_		2.5 [33]	2.2 at 369 nm [33]	1.42 for 7.2 nm thick [34]	0.62 [35]
	ReS_2_		~ 2.85 [36]	4.1 at 532 nm [37]	1.4 for 203 nm thick [38]	–
	PtSe_2_		~ 3.6 for holes [17]	–	~ 1.26 (Theoretical value) [39]	–
	GeAs		4.8 for holes [40]	4.4 at 830 nm [41]	2.9 (Theoretical value) [42]	–

**Fig. 2:**
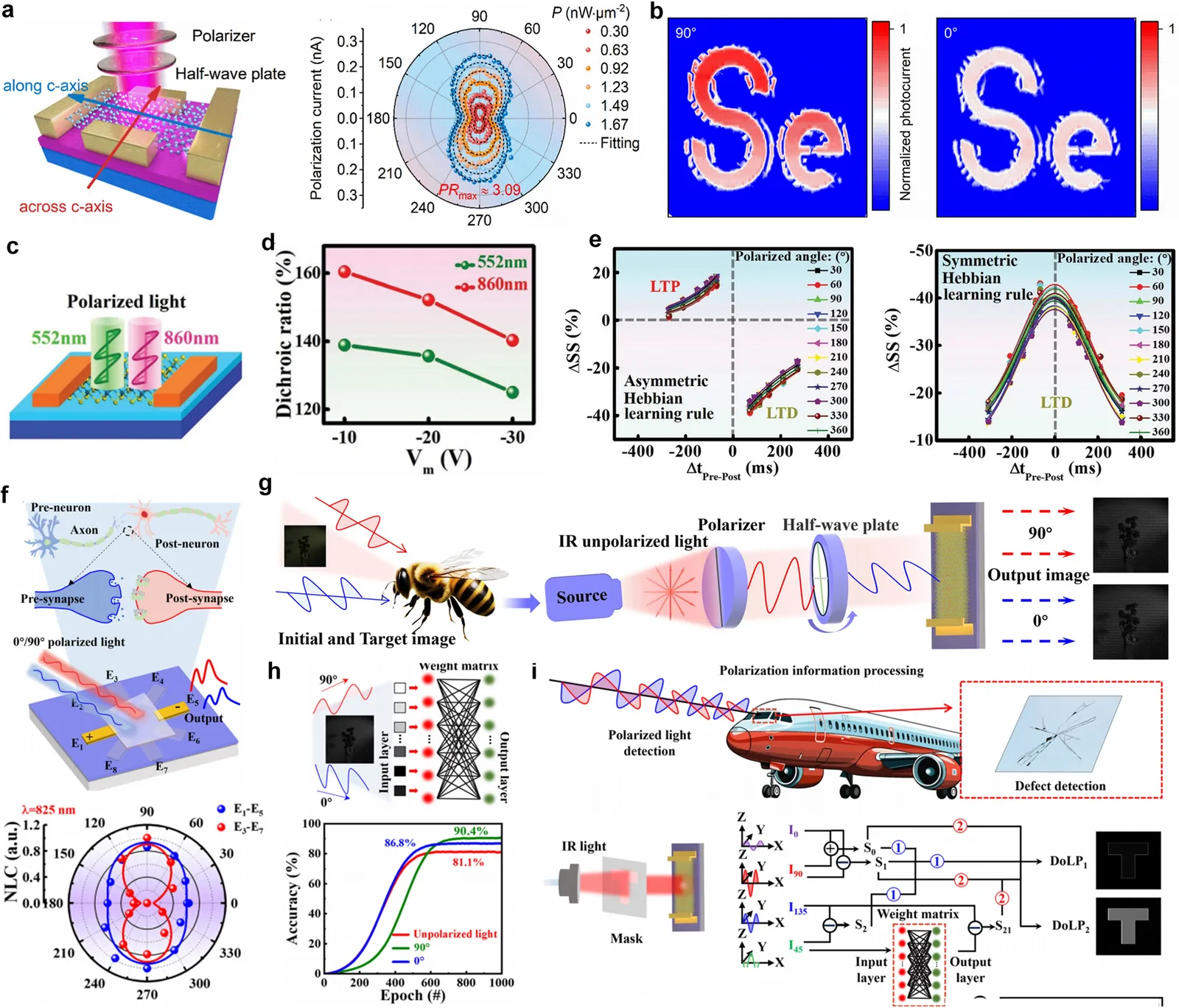
Anisotropic 2D materials enabled polarization-sensitive photodetectors and artificial synaptic devices. **a** Schematic illustration of the polarization-sensitive photodetector based on 2D Se and its anisotropic photoresponse under a 638 nm laser with various power densities. **b** Polarized imaging under 638 nm laser illumination with the patterns of “Se” at polarizing angles of 90° and 0°, respectively [46]. Copyright 2025, American Chemical Society. **c** Schematic diagram of artificial compound eyes based on ReS_2_ neuro-transistor. **d** Extracted excitatory postsynaptic current (EPSC) under different modulatory voltages plotted in the polar coordinates with different laser wavelengths of 552 nm and 860 nm. **e** Asymmetric and symmetric Hebbian STDP learning behavior under different polarization angles [45]. Copyright 2022, Royal Society of Chemistry. **f** Schematic diagram of the synaptic structure and synaptic behavior based on 2D ReSe_2_. **g** Schematic diagram of bees recognizing images under different polarized light directions and a simulation diagram of the image recognition. **h** Image recognition function of the device with image recognition accuracy for 0°/90° polarized directions. **i** Schematic diagram illustrating the flaw of hidden scratches on aircraft glass using polarized light for improved flaw detection information [11]. Copyright 2025, American Chemical Society

Among the diverse anisotropic properties exhibited by low-symmetry 2D materials, optical anisotropy holds particular significance for neuromorphic device engineering due to its ability to directly encode external visual information into synaptic weights without intermediate electrical conversion, thereby enabling intrinsically parallel and energy-efficient processing of optical signals [43]. Unlike conventional electrical synapses that rely solely on amplitude or timing modulation, polarization-based encoding offers inherent parallelism, wavelength selectivity, and direct compatibility with optical communication. Realizing this paradigm in hardware requires materials that exhibit strong and tunable anisotropic light–matter interactions. The intrinsically anisotropic crystal structures of 2D materials provide a direct physical substrate for mapping polarization information onto synaptic weights and learning rules. This section examines how the fundamental anisotropy of 2D materials can be harnessed not merely as a physical property to be characterized, but as a computational resource to implement polarization-encoded synaptic plasticity, thereby establishing a materials-to-function pipeline for next-generation neuromorphic vision systems. For instance, the divergent carrier mobilities along different crystallographic axes can be harnessed to create synaptic devices with inherent spike-timing-dependent plasticity (STDP) [44]. Furthermore, the strong linear dichroism allows for the construction of optoelectronic neurons where synaptic weight is directly modulated by the polarization state of incident light [45]. As shown in Fig. 2a, Peng et al. on elemental 2D selenium (Se) highlights its unique quasi-1D chain-derived anisotropy to fabricate photodetectors with a high polarization ratio (PR≈3.09) [46]. Furthermore, this work demonstrated polarization-dependent imaging by capturing distinct “Se” pattern contrasts at 0° and 90° polarization angles, directly visualizing the in-plane anisotropic photoresponse of their 2D selenium flake (Fig. 2b). This anisotropic optical response provides a direct hardware counterpart to biological polarization vision, enabling neuromorphic devices to natively process and distinguish complex optical information patterns without pre-conversion to electronic signals, which is pivotal for realizing ultra-low-power, parallel visual processing systems.

Xie et al. demonstrated a ReS_2_ neuro-transistor with remarkable wavelength-dependent reconfigurability in visual perception, fundamentally altering its anisotropic photoresponse with excitation transitions from visible (552 nm) to near-infrared (860 nm) illumination (Fig. 2c, d) [45]. More significantly, the device achieves unprecedented dynamic modulation of STDP learning rules, where both asymmetric and symmetric Hebbian learning behaviors are precisely governed by the polarization state of incident light (Fig. 2e). The underlying mechanism lies in the in-plane anisotropic band structure of ReS_2_, which results in polarization-dependent photogeneration rates and carrier mobilities. Specifically, the excitation of anisotropic excitons with distinct polarization orientations leads to their dissociation and transport being governed by directionally effective masses, thereby modulating the number and temporal profile of carriers reaching the channel. This directly influences the STDP timing window and the polarity of weight updates, linking optical polarization to synaptic learning rules. This breakthrough effectively encodes polarization information directly into neuromorphic computational algorithms, establishing a physical hardware platform that intrinsically links optical stimulus properties with adaptive learning capabilities.

Recently, Zhu et al. designed a ReSe_2_-based optoelectronic memristor with polarization angle-dependent synaptic plasticity, demonstrating the orientation-tunable dichroic responses attributed to defect-mediated charge trapping/detrapping at Se vacancy sites (Fig. 2f) [11]. It reveals that Se vacancies preferentially align along specific crystallographic directions in ReSe_2_, creating an anisotropic distribution of trap state densities. This asymmetric trap potential leads to direction-dependent rate constants for charge capture and emission. As the polarization angle of incident light varies, the absorption coefficient and exciton generation efficiency along different axes change, modulating the trap occupancy and ultimately yielding polarization-dependent synaptic plasticity. Such anisotropy-induced kinetics in trapping/detrapping dynamics underpins the observed dependence of conductance update characteristics such as linearity and symmetry on polarization orientation. To realize the integration of sensing and processing, the compound-eye-inspired polarized vision system was proposed to mimic hymenopteran optical communication via polarization-selective synaptic plasticity (Fig. 2g), where a high pattern recognition accuracy of 90.4% was achieved, exceeding unpolarized implementations by 9.3% (Fig. 2h). Finally, considering the application of polarization imaging technology in surface flaw detection, a polarization-engineered defect detection paradigm for aircraft glass was designed by harnessing the polarization-state perturbations caused by surface flaws under natural light to generate angle-dependent reflections that highlight defects (Fig. 2i).

Collectively, this progression from polarization-sensitive photodetectors to bio-inspired systems and plasticity-engineered memristors charts a promising pathway for polarization-perceptual neuromorphic hardware based on anisotropic 2D materials. However, the synaptic plasticity and memoristic behaviors in these single-material enabled devices are often fundamentally constrained by stochastic charge trapping at intrinsic or engineered defects, and this originates from the asymmetric spatial distribution of trap states within the anisotropic potential landscape, which gives rise to direction-dependent kinetics and fluctuations in trapping/detrapping processes, ultimately limiting the linearity, symmetry, and precision of conductance updates. Therefore, future integration of heterostructure engineering to introduce reliable memory mechanisms, synergistically combined with their intrinsic anisotropy, is poised to advance the development of high-performance neuromorphic devices with enhanced stability and controllability.

### Janus 2D Configurations

**Fig. 3:**
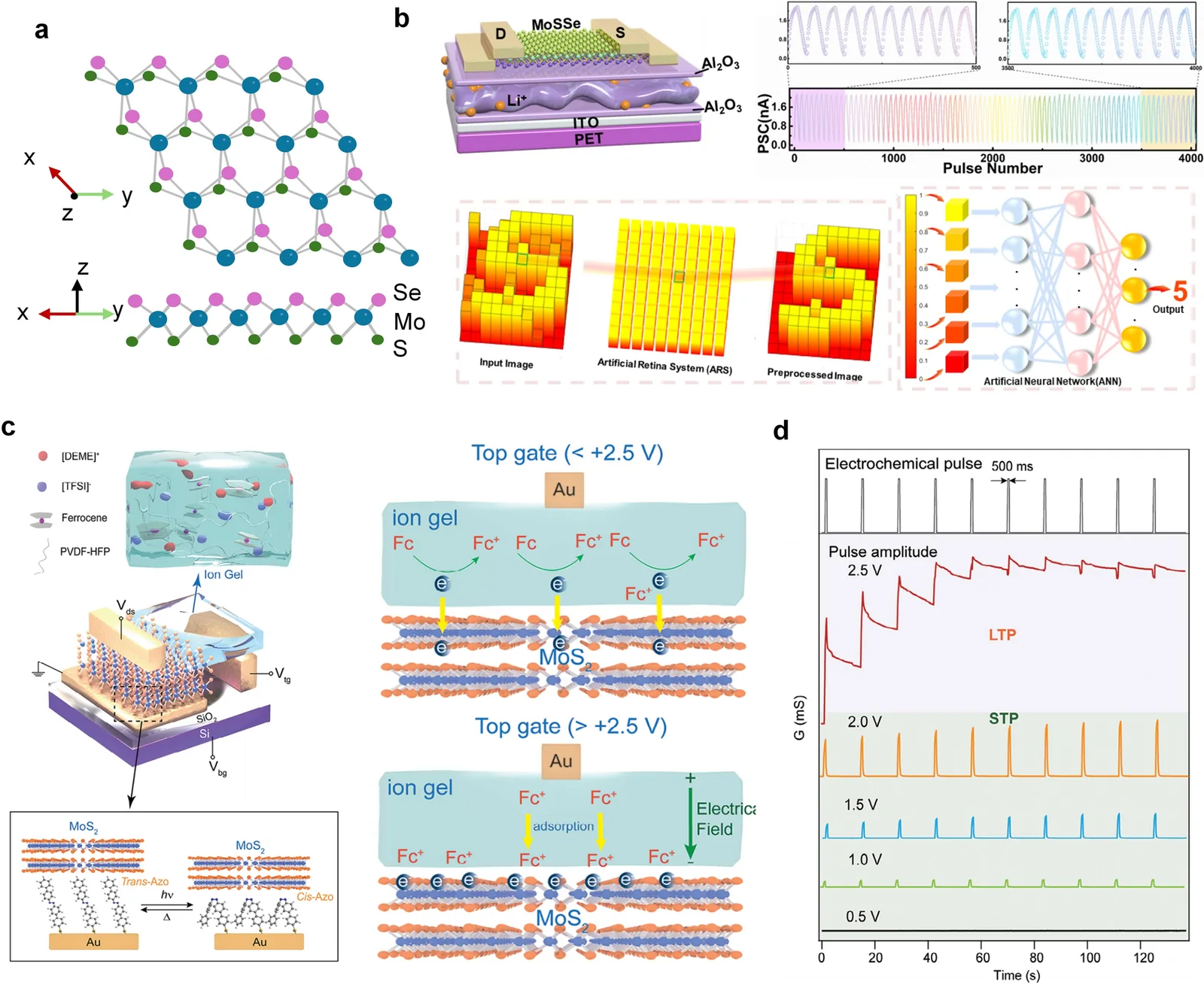
Artificial synaptic devices based on Janus 2D materials. **a** Schematic illustration of the Janus 2D MoSSe. **b** An optoelectronic synaptic transistor based on Janus MoSSe with typical long-term potentiation–depression (LTP/LTD) and its applications in artificial retina system to realize image recognition [50]. opyright 2022, American Chemical Society. **c** Schematic representation of device structure of Janus Fc/MoS_2_/Azo and the electrochemical charge transfer between Fc/Fc^+^ and MoS_2_ and adsorption of Fc^+^. **d** Transition between STP and LTP achieved by applying different electrochemical pulses [51]. Copyright 2023, WILEY–VCH

Janus 2D materials represent an emerging class of atomic-scale sheets characterized by their intrinsic structural asymmetry [14]. Unlike conventional 2D materials like graphene, a typical Janus monolayer, such as MoSSe, is constructed by sandwiching a transition metal layer between two different chalcogen layers as shown in Fig. 3a. This breaks the out-of-plane mirror symmetry, creating a polar axis perpendicular to the plane. This unique asymmetric structure directly gives rise to extraordinary physical properties such as a strong built-in electric field [47]. Such built-in field originates directly from the net dipole moment created by the electronegativity difference and structural disparity between the two distinct surface layers. This inherent polarization facilitates the creation of novel piezoelectric sensors and energy-efficient, atomically thin field-effect transistors without the need for external gating, enabling a new generation of low-power electronics [48].

Furthermore, the unique physical properties of Janus 2D materials position them as a highly promising platform for neuromorphic applications. The inherent built-in electric field that enables efficient photogenerated carrier separation and the strong spin–orbit coupling allowing for spin-manipulated photocurrent for non-volatile memory can be leveraged to modulate synaptic weight with high energy efficiency, emulating the behavior of biological synapses [49]. As shown in Fig. 3b, an artificial retina based on 2D Janus MoSSe was fabricated with Al_2_O_3_ as the trapping layer and Li^+^-based electrolyte as the functional layer [50]. This work demonstrates that the out-of-plane polarization and ion-coupling properties of Janus 2D MoSSe enable opto-electro-ionic multimodal co-modulation, allowing a single device to simultaneously achieve light perception, spike-timing-dependent plasticity, and preprocessing-recognition functions. Besides, Paolo et al. designed and fabricated a Janus MoS_2_ by asymmetrically functionalizing its two surfaces with a ferrocene (Fc)/ferrocenium (Fc^+^) redox couple and a photochromic azobenzene (Azo) self-assembled monolayer, enabling precise electrochemical doping and adsorption/desorption control of Fc/Fc^+^ species (Fig. 3c) [51]. By tuning the electrochemical stimulus magnitude as shown in Fig. 3d, the transition between volatile short-term plasticity (STP) and non-volatile long-term plasticity (LTP) is achieved, where lower potentials induce reversible charge transfer for STP, while higher potentials trigger Fc^+^ adsorption on MoS_2_ for LTP. Unlike traditional Janus 2D materials created via atomic substitution, this supramolecular engineering approach asymmetrically dresses the basal planes with distinct functional molecules, enabling dynamic and reversible multimodal synaptic plasticity without altering the intrinsic lattice structure.

More significantly, the piezoelectricity of Janus structure enables the direct conversion of mechanical stimuli into electronic signals, facilitating the development of self-powered, multimodal artificial synapses that can simultaneously process tactile and electrical information [52]. However, current research on piezoelectric tactile sensors primarily utilizes Janus structures based on fibers or composite systems [53], while the inherent and strong piezoelectricity of atomically thin Janus 2D materials themselves remains relatively under-explored for such biomimetic applications. Therefore, leveraging the intrinsic piezoelectricity of Janus 2D materials to fabricate ultrathin, direct neural-interfaced sensors represents a promising avenue for achieving highly sensitive.

### Symmetry-Broken 2D Ferroelectrics

**Fig. 4:**
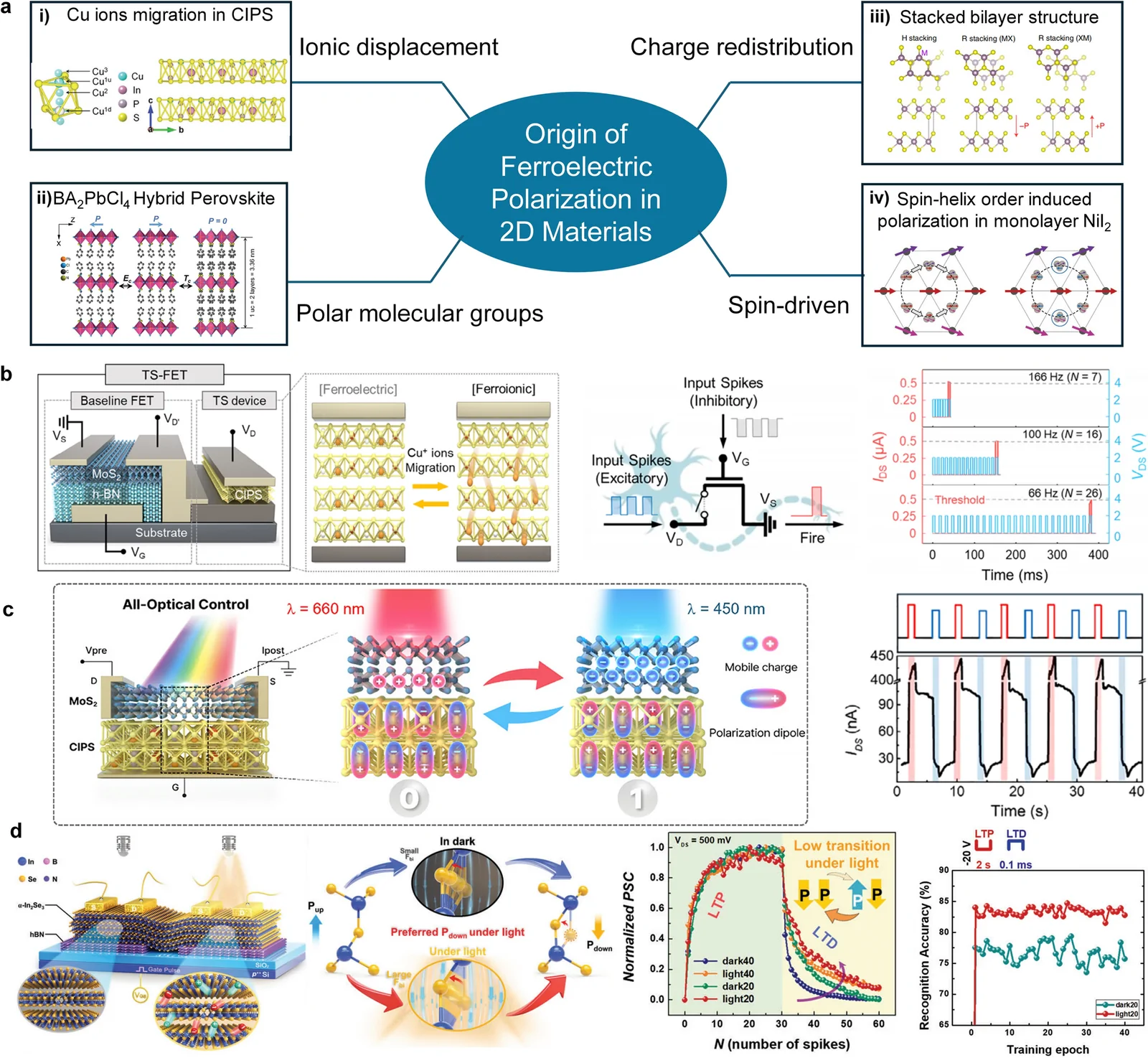
Applications of 2D ferroelectrics in neuromorphic devices. **a** Schematic illustration of the origin mechanism of polarization characteristics in 2D ferroelectric. i) Cu ions migration [63]. Copyright 2023, WILEY–VCH. ii) Hybrid perovskite [64]. Copyright 2018, WILEY–VCH. iii) Stacked bilayer structure [65]. Copyright 2022, Springer Nature. iv) Spin-helix order induced polarization [66]. Copyright 2022, WILEY–VCH. **b** Schematic of a ferroionic TS-FET and its emulation of a biological neuron. The device, integrating a MoS_2_ channel with a CIPS threshold switch, exhibits abrupt switching via Cu⁺ ion migration, enabling integrate-and-fire spiking dynamics that depend on input pulse intervals [58]. Copyright 2025, WILEY–VCH. **c** Structure of all-optically controlled neuromorphic device consisting of 2D ferroelectric heterostructure and its working principle for polarization switching [61]. Copyright 2026, The Author(s). **d** Schematic of the α-In_2_Se_3_-based FeFET under dark and light conditions, illustrating the light-enhanced built-in electric field and its influence on polarization transition. The resultant improvement in synaptic plasticity and recognition accuracy highlights the efficacy of photo-assisted modulation [62]. Copyright 2024, WILEY–VCH

The emergence of 2D ferroelectric materials is fundamentally rooted in symmetry breaking within the atomic lattice. Unlike their non-ferroelectric counterparts, these materials possess a non-centrosymmetric crystal structure, which is a prerequisite for spontaneous polarization. As summarized in Fig. 4a, the origins of spontaneous polarization in 2D ferroelectrics primarily encompass ionic-displacement-induced polarization, contributions from polar molecular groups, charge redistribution-induced polarization, and spin-driven polarization, contributing to the critical breaking of inversion symmetry [54]. Although multiple mechanisms often interact in real 2D systems such as CuInP_2_S_6_ (CIPS) [55] and α-In_2_Se_3_ [56], complicating the analysis, the resultant inversion symmetry breaking allows the polarization to be electrically switched and enables the essential non-volatility for memory devices.

This intrinsic non-volatile memory characteristic is pivotal for next-generation electronics, most notably for neuromorphic devices, where the analog-like switching of ferroelectric domains directly emulates synaptic weight modulation, enabling energy-efficient in-memory computing beyond the von Neumann architecture [57]. For neuromorphic devices, 2D ferroelectric materials can be broadly categorized by their functional role within the device architecture: as a dielectric layer, exemplified by CIPS, or as a conductive layer, represented by semiconducting α-In_2_Se_3_. Recently, Sungjoo et al. demonstrated an energy-efficient spiking neuron device based on a CuInP_2_S_6_ (CIPS) threshold-switching field-effect transistor (FET), which harnesses the tunable dynamics of Cu⁺ ion migration to induce an abrupt resistive transition as shown in Fig. 4b [58]. This CIPS-based neuron successfully emulates the essential leaky integrate-and-fire (LIF) behavior, enabling its seamless integration into a spiking neural network that achieves high recognition accuracy. Furthermore, CIPS has also been implemented as a gate dielectric in synaptic transistors [59] or resistive switching layer in memristors [60], enabling the construction of diverse synaptic devices. Sungjoo Lee et al. reported an all-optically controlled memristive device based on a van der Waals heterostructure of ferroelectric CIPS and semiconducting MoS_2_ as shown in Fig. 4c [61]. In this system, wavelength-dependent illumination modulates the interaction between photogenerated carriers and ferroelectric polarization charges, enabling bidirectional polarization switching in CIPS. The distinct carrier dynamics under different excitation wavelengths lead to opposite interfacial charge accumulation, thereby inducing opposite polarization states. This optically driven control of ferroelectric polarization introduces a tunable interfacial asymmetry in carrier distribution, which can be exploited to emulate excitatory and inhibitory synaptic plasticity. Such a mechanism highlights the important role of symmetry breaking mediated by optically controlled ferroelectric polarization in regulating carrier transport and neuromorphic functionalities in 2D material systems. Besides, Fig. 4d shows α-In_2_Se_3_-based synaptic transistor, where the light illumination cooperating with electrical gates tailored the ferroelectric polarization [62]. This photo-ferroionic synergy effectively improves the linearity of synaptic weight update, notably in LTD, thereby significantly enhancing the recognition accuracy in neuromorphic simulation. Overall, the intrinsic, non-volatile polarization in 2D ferroelectrics provides a direct and robust physical embodiment of synaptic weight, enabling the creation of energy-efficient neuromorphic devices with inherently memory-centric architectures.

## Structure Level: Engineered Interface Asymmetry

While exploiting intrinsic asymmetric properties of 2D materials provides a promising platform for neuromorphic computing, it often faces challenges in terms of precise modulation and device integration. To overcome these limitations, constructing artificial asymmetric structures, particularly heterojunctions, offers an alternative pathway [67]. This structural engineering approach enables precise manipulation of interface physics, such as carrier transport [68] and band alignment [69], which not only enhances device performance but also introduces additional degrees of freedom for functional tailoring. By harnessing such interfacial effects, rich neuromorphic behaviors, including synaptic plasticity and dynamic filtering, can be effectively emulated, highlighting the unique advantages of structural asymmetry in building advanced neuromorphic systems [70]. In this section, we reviewed the state-of-the-art neuromorphic devices engineered by the structure-level asymmetric design.

### Mixed-Dimensional Heterojunction

The construction of mixed-dimensional heterostructures (MDHs), which involve the integration of materials with different dimensionalities such as 2D/0D, 2D/1D, and 2D/3D, presents a powerful strategy for engineering asymmetry [71]. The inherent dimensional mismatch and the resulting interfacial effects, such as modified charge transfer and spatially confined band bending, create a pronounced built-in asymmetry. This pronounced asymmetry gives rise to a rich spectrum of tailorable physical phenomena, such as a photo-gating effect (PGE) that unifies perception and learning [72], and highly anisotropic charge transport engineered by incorporating intrinsically anisotropic 2D materials for implementing direction-sensitive neural dynamics [73]. These emergent properties of MDHs are highly conducive to emulating complex neurobiological behaviors, which are fundamental for building advanced neuromorphic systems [5].

**Fig. 5:**
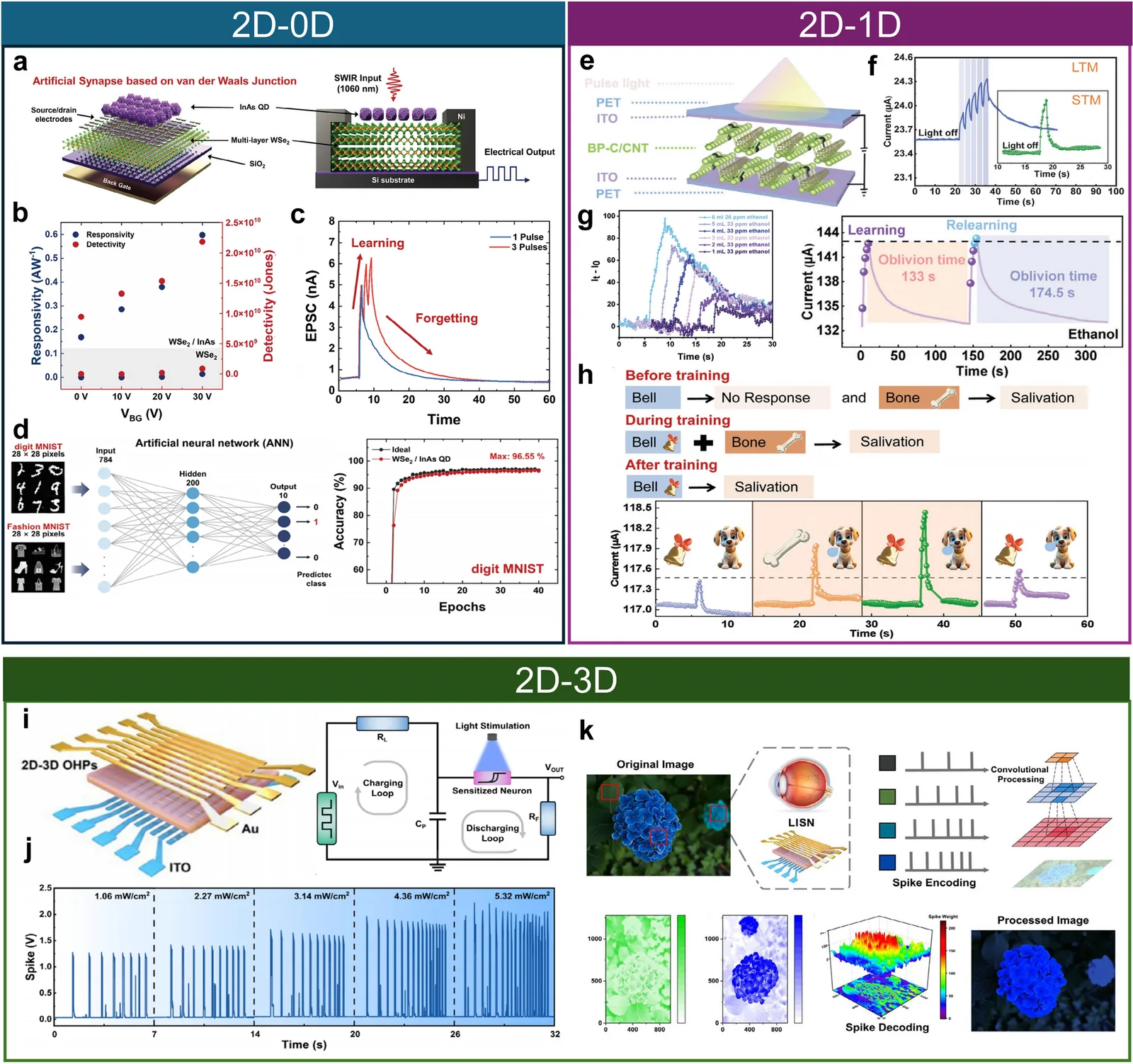
MDHs enabled neuromorphic devices. **a** Schematic illustration of a retinomorphic device based on 2D WSe_2_/0D InAs QDs MDHs. **b** Responsivity and detectivity of the bare WSe_2_ and WSe_2_/InAs FET under gate modulation. **c** Typical EPSC triggered by a single(blue) pulse and three (red) optical pulses to emulate the learning and forgetting behavior. **d** Schematic of ANN training for classification of the digit MNIST and the simulated recognition accuracy [75]. Copyright 2025, WILEY–VCH. **e** Schematic diagram of the artificial synapse devices constructed by the 2D BP/1D CNT hybrid filter membrane. **f** Transition from STM to LTM mode by increasing light pulse numbers. **g** Device response to ethanol gas at different volumes and the simulation of the “learn-forget-learn” function in ethanol gas. **h** Pavlov experiment diagram and simulation of Pavlovian conditioning experiments [77]. Copyright 2024, WILEY–VCH. **i** Schematic diagram of the Au/(PEA)_2_PbI_4_/MAPbI_3_/ITO 2D/3D hybrid memristor structure and its enabled circuit of LISN. **j** Firing characteristics of LISN at different light densities. **k** LISN-based color feature extraction of visual images [79]. Copyright 2025, WILEY–VCH

In 2D/0D mixed-dimensional heterostructures, the formed interface always facilitates efficient carrier separation, while the quantum confinement of 0D quantum dots (QDs) extends the spectral response range, which is a critical advantage for developing artificial vision systems [74]. Recently, Soobin et al. reported a retinomorphic artificial synapse device based on 2D WSe_2_/InAs QDs heterostructure as shown in Fig. 5a, where the quantum confinement effects of InAs QDs facilitated efficient short-wavelength infrared detection [75]. Notably, the InAs QDs components also worked as charge-trapping centers, inducing asymmetry in photogenerated electron–hole separation. The subsequent trapping of one type of carrier acts as a light-induced gate field, known as the PGE. This mechanism not only enhances the gate modulation on photoresponse (Fig. 5b) but also prolongs the carrier lifetime, thereby enabling fundamental memory characteristics that form the basis for synapse-like learning and forgetting operation (Fig. 5c). Furthermore, the simulation work demonstrated a high accuracy of ~ 96.55% in the digit MNIST dataset (Fig. 5d), underscoring the potential of integrating QDs with 2D materials to construct advanced retina-inspired neuromorphic devices. Besides, Ferrarese Lupi et al. designed a neuromorphic 2D memitter by integrating self-assembled plasmonic gold nanoparticles (AuNPs) with chemical vapor deposition (CVD)-grown monolayer WS_2_ flakes, which capitalized on the dimensional mismatch between 2D WS_2_ and 0D AuNPs [76]. Such memitter is an all-optical neuromorphic device capable of memorizing the history of optical stimulation and converting it into adaptive photoluminescence (PL) responses, which can emulate core functionalities such as short-term synaptic plasticity and visual memory. The MDH is vital for high-performance memitters, as AuNP-induced localized surface plasmon resonance (LSPR) enhances light–matter interactions, boosts PL intensity while preserving the adaptive PL dynamics of WS_2_, and accelerates potentiation–depression kinetics, enabling a scalable, high-efficiency all-optical neuromorphic platform.

Distinct from 2D/0D system, the 2D/1D heterostructure such as 2D BP/1D carbon nanotube (CNT) (Fig. 5e) forms anisotropic charge transport pathway, which enable efficient carrier separation and stable interfacial charge trapping [77]. This asymmetric design underpins the transition from short (STM)- to long-term memory (LTM) under optical stimulation via a persistent PGE (Fig. 5f). More interestingly, the device exhibited synergistic multi-sensory integration, where gas adsorption modulates carrier density, while optical and electrical stimuli cooperatively tune synaptic weight (Fig. 5g). Furthermore, the heterostructure successfully emulates associative learning in a Pavlovian paradigm (Fig. 5h), demonstrating its capability to process and retain complex multimodal information for advanced neuromorphic functions. While effective, 2D/0D and 2D/1D systems face constraints in achieving the functional complexity and environmental stability offered by 2D/3D heterostructures, which provide a more robust and versatile platform [78]. Figure 5i demonstrates a 2D–3D organic–inorganic hybrid perovskites (OHPs) memristor which, by leveraging its tunable decay and wavelength selectivity, enabled the development of a light-induced sensitized neuron (LISN) with enhanced firing frequency through fundamental circuit design [79]. Such 2D/3D heterostructure contributed to optically regulated neuronal spiking behavior by modulating the heterojunction potential barrier through photogenerated carriers, which effectively tuned the spike frequency in response to varying light intensities (Fig. 5j). Owing to the modulation of pulse frequency and rate of change by wavelength, as-fabricated LISN was able to emulate the color-perception mechanism of biological visual systems (Fig. 5k). In summary, the asymmetric structure of MDHs provides a powerful and versatile mechanism for tailoring device response characteristics, underscoring their significant advantage in the design of multifunctional neuromorphic systems.

### Band Alignment Engineering

**Fig. 6:**
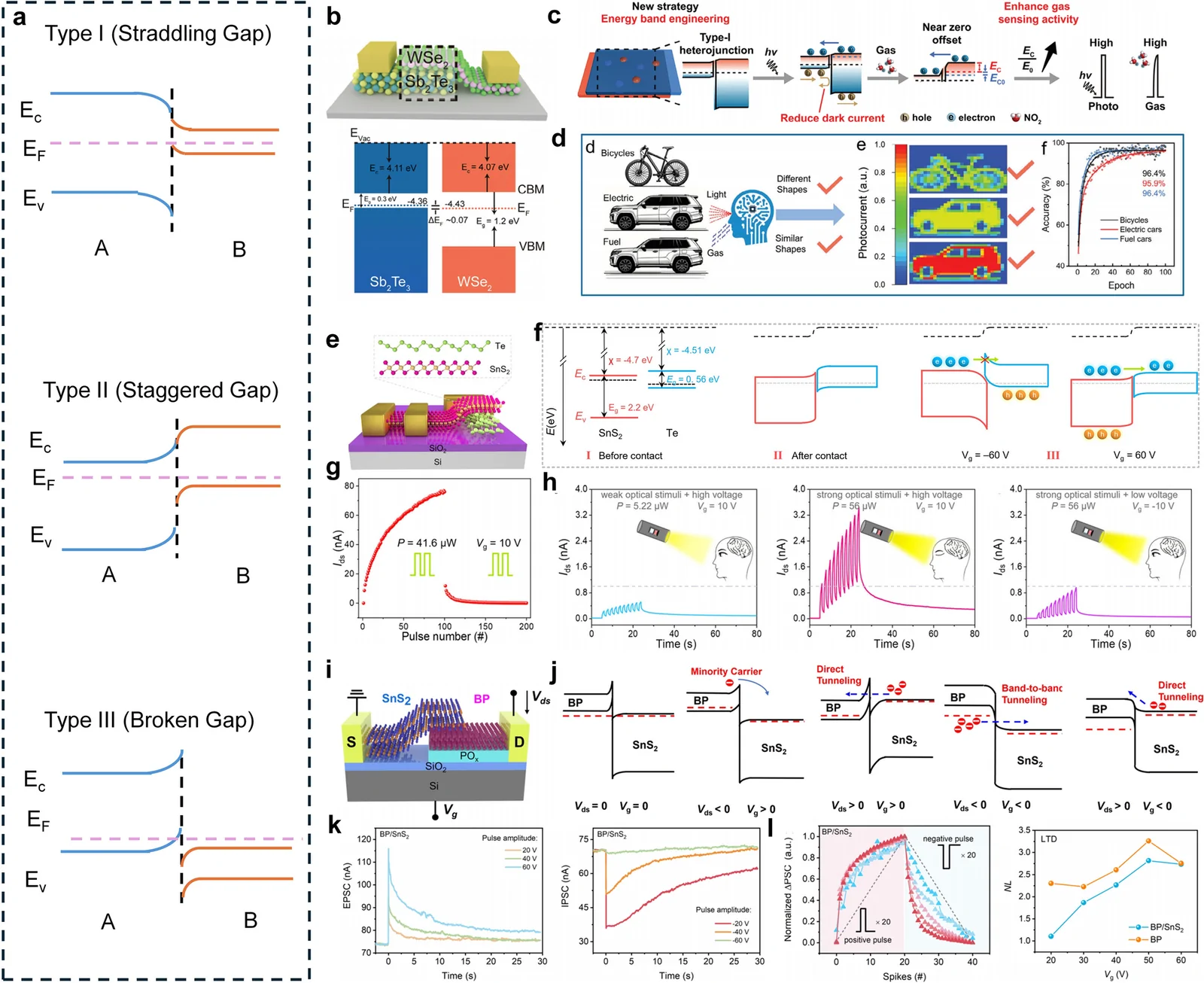
Engineered band alignment in neuromorphic devices based on 2D vdWs heterojunctions. **a** Schematic illustration of three typical band alignment in 2D vdWs heterojunction. **b** Schematic image of the Sb_2_Te_3_/WSe_2_ heterojunction device and their band structure before contact. **c** Scheme illustration of the Type I heterojunction design strategy for high light on/off ratio photodetector and high-sensitivity gas sensor. **d** Vision-olfaction dual-mode recognition, along with simulated sensory perception images [86]. Copyright 2024, WILEY–VCH. **e** Schematic diagram of SnS_2_/Te vdWs heterojunction. **f** Schematic band structure under the modulation of *V*_*g*_. **g** Optical potentiation and electrical depression characteristics. **h** Demonstration on the light adaptation of the device [87]. Copyright 2025, WILEY–VCH. **i** Schematic of the BP/SnS_2_ heterostructure device. **j** Energy band alignment under different voltage configurations. **k** EPSC and IPSC triggered by the opposite voltage pulse amplitudes. **l** LTP/LTD behaviors and the calculated nonlinearity with respect to different voltage amplitudes [88]. Copyright 2025, American Chemical Society

In contrast to the geometric asymmetry of MDHs, vdWs heterostructures composed entirely of 2D materials exhibit intrinsic asymmetry primarily through their band alignment. As illustrated in Fig. 6a, three representative band alignment configurations including Type I (Straddling Gap), Type II (Staggered Gap) and Type III (Broken Gap) demonstrate this inherent asymmetric characteristic. The band structure of 2D materials is not only material-dependent but also exhibits strong layer-thickness sensitivity [80–82]. This tunability enables precise engineering of band alignment in vdWs heterostructures, thereby effectively modulating carrier transport behaviors and opening avenues for developing neuromorphic devices with diverse functionalities [83–85].

In a Type I band alignment, the smaller-bandgap semiconductor is energetically nested within the conduction- and valence-band edges of the larger-bandgap one, exhibiting an intrinsic asymmetry wherein both carrier confinement potentials are directed toward the same layer due to the simultaneous downward shift of its conduction-band minimum and upward shift of its valence band maximum relative to the adjacent semiconductor. As shown in Fig. 6b, a vdWs heterojunction contrasted by smaller-bandgap Sb_2_Te_3_ and larger-bandgap WSe_2_ was fabricated with a typical Type I band alignment, which demonstrated the integrated photoelectric and gas sensing performance in a single device (Fig. 6c) [86]. The simultaneous realization of such dual-mode sensing could be attributed to the fact that the single-side valence-band barrier of the Type I band alignment suppressed dark current for enhanced photoresponse, while its near-zero conduction-band offset was leveraged to amplify gas-induced charge transfer perturbations for highly sensitive and selective gas detection. The asymmetric potential profile of the Type I band alignment directly governs carrier dynamics. The valence-band barrier selectively suppresses hole injection/extraction, enhancing the separation efficiency of photogenerated electron–hole pairs, while the near-zero conduction-band offset facilitates electron transfer and amplifies charge transfer perturbations induced by gas adsorption. Moreover, this asymmetric barrier shapes the energy distribution of trap states at the interface, leading to improved linearity in conductance updates by modulating the field-dependent capture/emission rates. Benefiting from these synergistic properties, the heterojunction achieves biomimetic visual–olfactory dual-mode recognition within a single device, boosting the accuracy of distinguishing electric and fuel-powered vehicles from ~ 50 to ~ 96% (Fig. 6d).

Distinct from the carrier-confined Type I configuration, a Type II band alignment places the conduction-band minimum and valence-band maximum in different semiconductors, creating a pronounced interlayer asymmetry that drives directional electron–hole separation and establishes a built-in potential gradient across the heterointerface. A Type II SnS_2_/Te vdWs heterojunction provided an energetically staggered band configuration that spatially separated photogenerated carriers, allowing optical stimuli to modulate channel conductance with high efficiency (Fig. 6e, f) [87]. When combined with gate-voltage control, this interlayer asymmetry enables synergistic tuning of excitatory and inhibitory synaptic responses, thereby emulating key neuromorphic functions (Fig. 6g). The staggered band alignment creates an internal electric field that spatially separates electrons and holes, extending carrier lifetimes and enabling efficient optical modulation of channel conductance. The gate voltage further tunes the band bending, altering the overlap of wavefunctions and the effective barrier for carrier recombination or trapping. This dual modulation allows precise control over the kinetics of trap filling and emptying, which directly dictates the linearity and symmetry of excitatory and inhibitory synaptic responses. The adaptability to different light intensities, as demonstrated by the visually comfortable state, arises from the gate-tunable asymmetry of the potential profile, which compensates for variations in photogenerated carrier density. As shown in Fig. 6h, this dual-modulation pathway enhanced plasticity programmability and made it adaptive to the visually comfortable state like human eye under strong light by adjusting *V*_*g*_.

Defined by a broken-gap configuration where the conduction-band minimum of one semiconductor drops below the valence-band maximum of the other, the Type III heterojunction exhibits a pronounced asymmetry that originates from this inverted band ordering, enabling cross-band carrier transfer and interlayer tunneling, which is not available in Type I or Type II band structure. Recently, Lv et al. demonstrated an artificial synaptic device based on a BP/SnS_2_ vdWs heterojunction (Fig. 6i) [88]. Figure 6j illustrates the gate-voltage-modulated carrier tunneling mechanisms within its Type III band alignment where the Fermi-level shift under different gate biases effectively switched between band-to-band tunneling and direct tunneling, enabling versatile and tunable synaptic conductance updates. In the broken-gap alignment, the inverted band ordering enables interlayer tunneling, which is highly sensitive to the applied gate bias. The Fermi-level shift modulates the tunneling mechanism from band-to-band tunneling to direct tunneling. This tunable tunneling kinetics results in linear and symmetric conductance updates because the tunneling probability can be engineered to vary linearly with gate voltage over a certain range. Additionally, trap-assisted tunneling processes at the interface may contribute to the superior linearity observed in long-term depression, as the asymmetric potential confines carriers and enhances the uniformity of charge redistribution. Finally, this device demonstrated the transition from excitatory to inhibitory plasticity by modulating gate pulse polarity (Fig. 6k), while the heterostructure interface enabled superior linearity in long-term depression, which was crucial for high-accuracy neural network training (Fig. 6l).

In fact, band alignment is not static but undergoes dynamic reconfiguration under external stimuli such as applied voltage, forming the foundation for achieving diverse neuromorphic functionalities through band alignment engineering. This dynamic reconfiguration directly impacts carrier capture and release kinetics because applied voltages modify the potential profile and in turn alter trap depths, barrier heights, and tunneling probabilities. These changes further determine the temporal evolution of conductance such as the linearity or symmetry and precision of synaptic weight updates. By engineering the band alignment and its response to external stimuli, one can achieve mechanistic control over the underlying physical processes that govern neuromorphic performance. Moreover, the aforementioned band alignment configurations are not limited to 2D vdWs heterostructures but are equally applicable for analyzing other heterojunctions such as MDHs.

### Surface and Interface Engineering

The emerging strategy of constructing asymmetric structures within a single 2D material system via localized surface and interface modification presents a compelling avenue for neuromorphic engineering. This approach fundamentally relies on tailoring local properties, for example, through selective doping or defect engineering, to create built-in fields and electronic asymmetry [89–91]. This site-specific engineering avoids the lattice mismatch of heterostructures, thus enabling precise spatial modulation of conductance and switching dynamics within a homogeneous medium. In addition, surface adsorbates commonly present on exposed 2D materials can act as another form of local surface modification. They modify the interfacial potential and carrier distribution, thereby further introducing asymmetry in the device response [92, 93]. Such structural and interfacial asymmetry is crucial for emulating neurobiological functions, including directional signal propagation and various synaptic learning rules [94]. Consequently, this methodology establishes a robust and scalable platform for developing high-performance neuromorphic devices, unifying the required functional complexity with structural simplicity and integration compatibility.

**Fig. 7:**
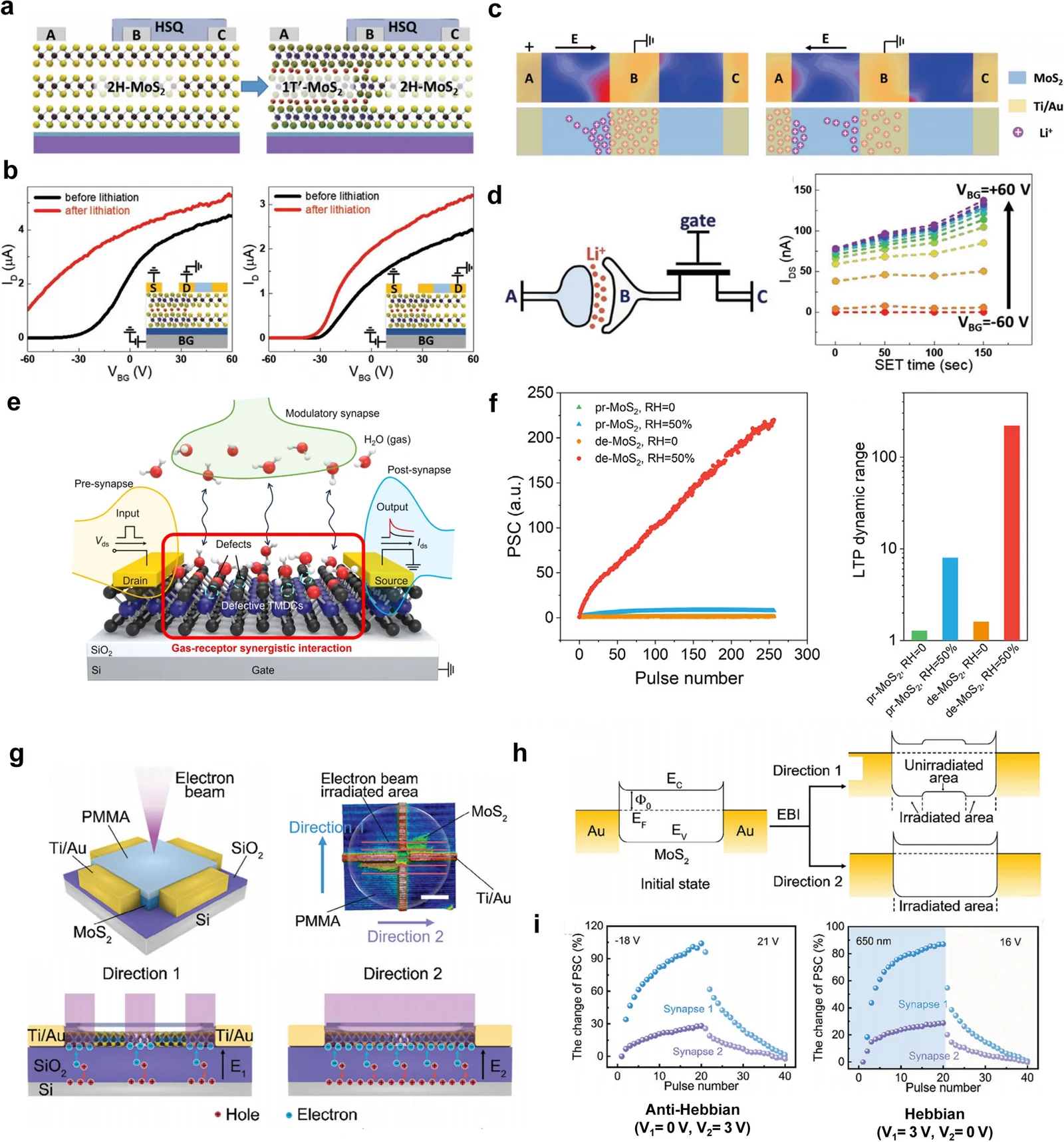
Neuromorphic devices based on 2D homojunctions achieved by local modification. **a** Schematics of the fabricated device before (left) and after (right) selective lithiation. **b** Transfer characteristics of 1 T’-only MoS_2_ (left) and 2H/1 T’-hybrid MoS_2_ devices (right) before and after lithiation. **c** Raman maps of the 1 T’-MoS_2_ phonon peak in LRS and HRS (top) and corresponding schematic images (bottom) representing the distribution of Li^+^ ions. **d** Schematic of the synapse-like MoS_2_ homojunction FET and its output current acquired at varying V_BG_ and the RS status [98]. Copyright 2020, WILEY–VCH. **e** Schematic of the GRSI mechanism in 2D TMDC memristors. **f** LTP and LTD measurements of the pr-MoS_2_ device and the de-MoS_2_ device and the extracted LTP dynamic ranges [99]. Copyright 2025, Elsevier Inc. **g** Schematic of the device n-type doping treatment procedures by electron beam. Synapse 1 and synapse 2 are represented by devices along direction 1 and direction 2, respectively. **h** Energy band diagrams of the MoS_2_ transistor before and after EBI. **i** Conductance modulation of the device for synapse 1 and synapse 2 under electric stimuli (left) and optical/electric stimuli (right), respectively [100]. Copyright 2024, WILEY–VCH

Given the numerous works covering homojunctions formed through local modification [95–97], this section focuses on recent progress in surface and interface engineering through adsorbates or controlled doping to construct asymmetric electronic structures within a single 2D material system for neuromorphic devices. Figure 7a illustrates an asymmetric MoS_2_ homojunction via chemical lithiation, where the device is immersed in n-butyllithium to locally convert the semiconducting 2H phase of MoS_2_ into a metallic 1 T' phase, which was further demonstrated by the comparison of transfer curves before and after lithiation (Fig. 7b) [98]. This phase configuration creates a dynamic Schottky barrier, enabling non-volatile, multi-level resistive switching through electric field-driven redistribution of the pre-intercalated Li^+^ ions (Fig. 7c). The controllable migration of these Li^+^ ions directly modulate the channel conductance, emulating essential synaptic behaviors such as a multi-state computing window with a higher degree of freedom achieved by electrostatic gating (Fig. 7d).

Surface adsorbates commonly present on exposed 2D materials can introduce pronounced electronic asymmetry and significantly influence device operation. Adsorbed molecules modify the local surface potential and carrier density through charge transfer. When adsorption is spatially non-uniform, asymmetric carrier distributions and built-in electric fields emerge, providing an alternative route to implement neuromorphic functionalities. Zhao et al. demonstrated that adsorbate-induced asymmetry can be deliberately engineered to enhance neuromorphic device performance [99]. They introduced defects into 2D MoS_2_ as receptors that strongly bind H_2_O molecules (Fig. 7e). This gas–receptor synergistic interaction (GRSI) creates pronounced electronic asymmetry, where charge transfer from adsorbed H_2_O to the defective lattice reversibly dopes the channel, enabling voltage-controlled switching between high- and low-resistance states with ratios exceeding 10^4^. Unlike passive adsorption effects, this mechanism leverages defect-engineered surface chemistry to achieve dynamic, tunable asymmetry. The resulting devices emulate both homosynaptic and heterosynaptic plasticity with large dynamic range (> 200) (Fig. 7f). This work establishes adsorbate-defect coupling as a designable principle for introducing functional asymmetry in 2D neuromorphic systems.

In addition to the effects of the above chemicals, material properties can also be modified via localized selective irradiation. This technique, as illustrated in Fig. 7g, utilized region-specific electron beam irradiation on a MoS_2_ surface to create trap sites and generated spatial conductance anisotropy through crystallographic orientation variations [100]. The anisotropic characteristics originated from the localized doping contrast between electron beam-irradiated and unirradiated regions, induced by electron–hole pair generation in the SiO_2_ layer, which causes a Fermi-level shift toward the conduction band in the irradiated areas as shown in Fig. 7h. Consequently, the periodic n–n⁺ junctions in Synapse 1 resulted in limited conductivity, in contrast to the highly enhanced conductivity of Synapse 2, which featured parallel conductive stripes. From Fig. 7i, the larger PSC change observed in Synapse 1 compared to Synapse 2 under both electrical (left panel) and photonic-potentiation/electrical-habitation stimuli (right panel) could be attributed to its limited conductance, which provided a wider dynamic range for modulation.

## Device Level: Extrinsic Geometric Asymmetry

Having discussed the significance of asymmetric design at the material and structure levels for enabling neuromorphic behaviors, this section now shifts the focus to the device level, where asymmetric configurations and their corresponding performance modulation strategies will be examined. This approach not only capitalizes on the advantages of material- and structure-level designs but also offers unique capabilities for system-level integration and dynamic control of device performance. Since neuromorphic devices are primarily categorized structurally into two-terminal memristors and three-terminal synaptic transistors, the following discussion will accordingly elaborate on asymmetric design strategies and performance modulation within these two dominant device architectures.

### Asymmetric Diffusive Memristor

**Fig. 8:**
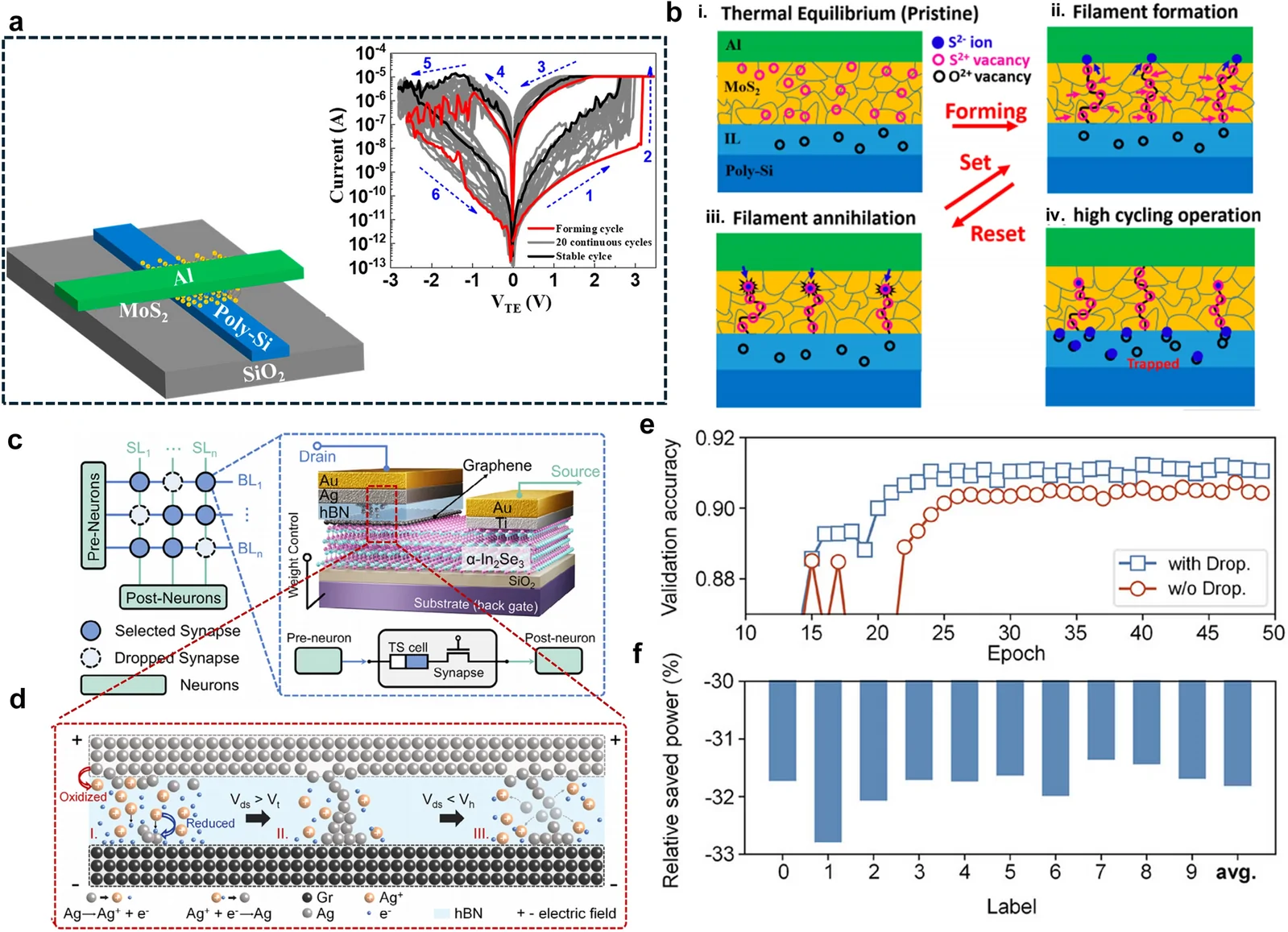
Asymmetric diffusive memristor. **a** Schematic of the memristor based on Al/MoS_2_/Poly-Si and its *I-V* sweeping characteristics. **b** Schematics showing the corresponding sulfur ions and vacancies distribution in different conditions [103]. Copyright 2024, American Chemical Society. **c** An illustration shows a DropConnect hardware implementation of the synaptic devices with self-drop capability. **d** Threshold switch resistance states corresponding to the five different states under positive bias conditions. **e** Evaluation accuracy of SpikingViT with or without DropConnect unit. **f** Relative saved power consumption for each category [104]. Copyright 2025, WILEY–VCH

Memristors, as non-volatile memory devices whose resistance states depend on the history of applied electrical stimuli, have emerged as fundamental building blocks for neuromorphic computing due to their inherent ability to emulate synaptic weight updates [101]. Among various types, diffusive memristors distinguish themselves by utilizing the controllable diffusion of metal ions or atoms within a switching medium to achieve volatile conductance switching, thereby effectively mimicking the temporal dynamics and decay characteristics of biological synapses [102]. To precisely control these spatiotemporal ion dynamics, asymmetric design strategies are critical. 2D materials and their heterostructures offer unparalleled advantages for fabricating asymmetric diffusive memristors. Their atomically flat interfaces and tunable interlayer coupling enable precise spatial confinement and anisotropic modulation of metal ion diffusion pathways and kinetics, while the ultrathin thickness and high carrier mobility boost the switching speed and conductance tuning precision of devices. Figure 8a presents an asymmetric diffusive memristor based on Al/MoS_2_/Poly-Si, where the asymmetry of S ion/vacancy diffusion and migration contributed to the bipolar resistive switching behaviors [103]. Additionally, the *I-V* sweeping measurements further revealed its asymmetric bipolar resistive switching behavior with the current rising abruptly under positive bias (set process) due to the rapid aggregation of sulfur vacancies into conductive filaments, while decreasing gradually under negative bias (reset process) as sulfur ions diffused back slowly to annihilate the filaments, reflecting the asymmetric dynamics of ion migration in the device. From Fig. 8b, a detailed illustration of the polarity-dependent diffusion process of sulfur ions and vacancies in the as-fabricated memristor was presented, where sulfur vacancies aggregated along grain boundaries to form filaments under positive bias (ii), and sulfur ions recombined with vacancies to break the filaments under negative bias (iii), with the incomplete annihilation of filaments in high-cycle operation (iv). This work reveals that engineering the asymmetric diffusion of ions/vacancies via grain boundary and interfacial layer modulation in 2D diffusive memristors provides a key design principle for mimicking biological synaptic asymmetry, offering critical guidance for developing high-performance neuromorphic devices.

Besides, recent systems-oriented works have demonstrated how asymmetric ion migration in 2D-based devices can directly address network-level challenges in neuromorphic computing. For example, Yu et al. demonstrated a threshold switch based on an Ag/h-BN/graphene heterostructure integrated with an α-In_2_Se_3_ ferroelectric field-effect transistor to implement hardware-level DropConnect regularization (Fig. 8c) [104]. The non-volatile memory effect in this device originates from the asymmetric diffusion behavior of Ag ions within the 2D layered h-BN (Fig. 8d). Under positive bias, Ag^+^ ions penetrate the h-BN stack to form conductive filaments, while upon voltage removal, the filaments spontaneously rupture due to the atomically pristine nature of h-BN and the chemically inert graphene electrode, which promotes spontaneous filament dissolution. Crucially, the intrinsic cycle-to-cycle variation in threshold voltage enables the threshold switch to function as a built‑in random number generator. This intrinsic stochasticity, arising from the probabilistic nature of Ag filament formation/dissolution in the 2D heterostructure, allows each synaptic connection to be randomly dropped during network training without any auxiliary circuits. At the system level, this hardware‑implemented DropConnect effectively combats overfitting in deep neural networks. When deployed in a spiking vision transformer for MNIST classification using only 2% of the training data, the network achieved a 1.36% accuracy improvement (Fig. 8e) and ~ 31.7% energy reduction (Fig. 8f) compared to DropConnect-free implementations. This exemplifies how the asymmetric, stochastic ion dynamics inherent to 2D heterostructures can be harnessed to address the critical network-level bottleneck of overfitting while enhancing energy efficiency. In another bio‑inspired design, a visual neuron transistor employing a MoSe_2_/MoS_2_ heterojunction channel integrated with parallel Al_2_O_3_‑based threshold switches demonstrated how asymmetric device configurations can enable robust population coding [105]. It shows that intrinsic variability in ion-based diffusive memristors can be transformed into a computational advantage at the network level, enabling more reliable and biologically inspired neuromorphic processing.

Collectively, these advances demonstrate that the unique weight update properties of 2D-based asymmetric diffusive memristors, particularly intrinsic stochasticity and population-level statistical averaging, directly address persistent network-level bottlenecks. The inherent randomness of ion migration dynamics enables hardware-native regularization that mitigates overfitting without peripheral overhead, while integrating multiple asymmetric units converts device variability into enhanced encoding fidelity and fault tolerance. By leveraging atomically precise interfaces and tunable ion kinetics in 2D heterostructures, these design principles establish a direct bridge between device physics and system-level computational efficiency, offering a scalable pathway toward robust learning models and biologically plausible neural encoding in next-generation neuromorphic hardware.

### Asymmetric Contact Engineering

Compared to memristors, field-effect transistors (FETs) offer greater tunability for the realization of neuromorphic behaviors. This advantage of FETs is evident in both structural and functional aspects. Structurally, they emulate a biological synapse by employing the gate electrode as the presynaptic terminal, the gate dielectric as the synaptic cleft, and the channel as the postsynaptic terminal. Functionally, their architectural design enables the incorporation of diverse technological approaches, including gate dielectric, contact, and channel engineering, which offer extensive degrees of freedom for tailoring synaptic plasticity [106–110]. In particular, asymmetric device design, which introduces structural asymmetry into the gate and channel, enables performance customization and offers a promising pathway for developing multifunctional neuromorphic devices. This section will primarily focus on 2D neuromorphic devices based on asymmetric contact engineering. Asymmetric contacts mainly arise from two strategies: the use of different metals for the two contacts [111, 112] and geometric asymmetry in the channel region [113, 114]. These asymmetric configurations lead to differentiated energy barriers at the interfaces between the channel and the two electrodes. Such asymmetric barrier characteristics are often exploited to achieve rectifying behavior and self-powered photoresponse, which are crucial for realizing low-power neuromorphic devices [115–117].

**Fig. 9:**
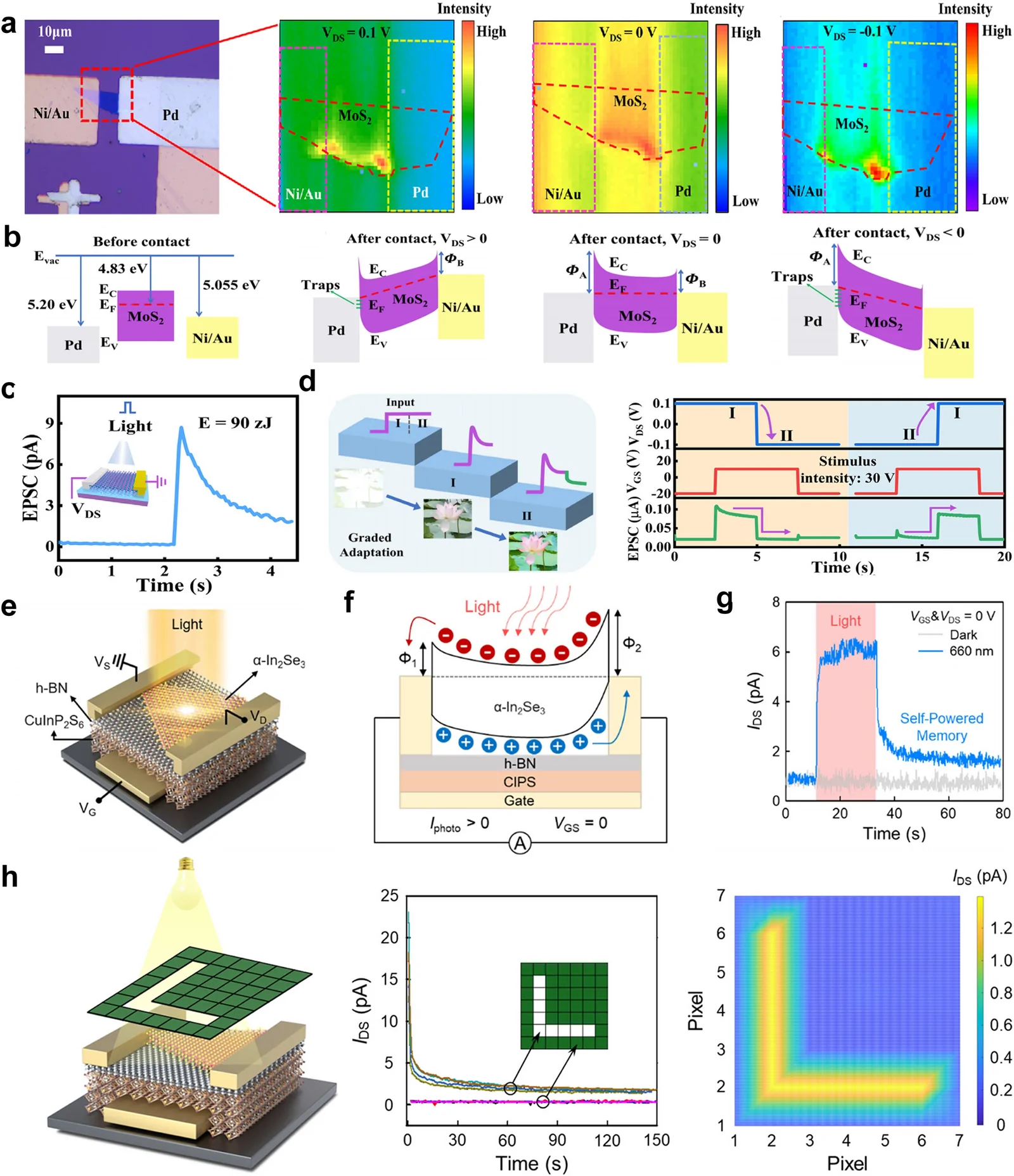
Neuromorphic FETs with asymmetric contacts. **a** Optical image of the MoS_2_ device with asymmetric Schottky barrier and its spatially resolved photocurrent mapping at different *V*_*DS*_. **b** Schematic energy band diagrams of the MoS_2_ FET under different conditions. **c** Synaptic event triggered by light pulse with ultra-low power consumption. **d** Schematic of the graded adaptation process achieved with switching channel operations [118]. Copyright 2025, AIP Publishing. **e** Structural diagram of the α-In_2_Se_3_-based FET with asymmetric contact areas. **f** Schematic energy band diagrams under illumination. **g** Temporal response of *I*_*DS*_ under dark and illumination at *V*_*GS*_ = 0 V and *V*_*DS*_ = 0 V.** h** Schematic illustration of real-time object recognition and reconstructed object image from the *I*_*DS*_ vs time curves [12]. Copyright 2025, American Chemical Society

The atomically thin nature of 2D materials is particularly advantageous here, as it allows for precise control over these interfacial barriers, making them highly susceptible to engineered asymmetry. Figure 9a presents an asymmetry-Schottky-barrier MoS_2_ phototransistor contacted by Pd and Ni/Au, respectively [118]. The spatially resolved photocurrent mapping (SRPM) dynamically captures how the photoresponse shifts from localized bright spots at the Pd contact (photovoltaic effect) under zero/reverse bias to a distributed current across the channel (photoconductive effect) under positive bias. This visually observed, bias-dependent evolution governed by asymmetric Schottky contacts could be further clarified by the corresponding energy band diagrams under different conditions (Fig. 9b), providing a crucial pathway for designing reconfigurable and low-power neuromorphic vision devices. Furthermore, the difference in Schottky barrier heights between the two contacts enables the device to generate a net photocurrent under zero or minimal bias, due to their distinct carrier separation efficiencies. As illustrated in Fig. 9c, the energy consumption of each synaptic event was determined to be approximately 90 zJ (9 × 10^20^ J) under extremely low read voltages (*V*_*DS*_ = 0.24 μV). Combined with *V*_*ds*_-induced reconfigurable synaptic behaviors, a low-energy bionic vision system with graded adaptation process was achieved by defining two modulation channels, I and II, referring to the positive and negative *V*_*ds*_ bias, respectively (Fig. 9d). This is achieved via a dual-channel synergistic modulation strategy: Under a constant stimulus, switching to the low-sensitivity channel (II) triggers rapid desensitization to prevent oversaturation from strong inputs, while switching to the high-sensitivity channel (I) provides immediate amplification to enhance weak signals. This intelligent transition allows the device to flexibly interpret signals of varying intensities, effectively emulating the adaptive behaviors of biological sensory neurons in dynamic environments.

Another attempt to realize the asymmetric contact between 2D materials and electrodes is to induce the different contact areas as shown in Fig. 9e, where a triangular α-In_2_Se_3_ possessed a different contact geometry with Au electrodes [12]. The formation of built-in potential was due to the difference of work functions, in which the work function with large contact area was larger, and the work function with small contact area was smaller (Fig. 9f). By this way, an optical memory behavior could be obtained under zero bias after light illumination (Fig. 9g), which laid the foundation of the visual perception in the retina. Figure 9h demonstrates the successful optical memory and retrieval of an “L”-shaped pattern at zero bias, achieved by sequentially recording and erasing each pixel’s photoresponse through pulsed illumination and gate-controlled reset operations. In summary, introducing asymmetric contact offers a powerful means to tailor interfacial energy barriers, modulate carrier dynamics, and thus encode rich synaptic functionalities directly within the device physics. This approach not only enables essential features such as rectification, self-powered operation, and ultra-low power consumption but also facilitates the design of reconfigurable and adaptive systems capable of processing complex, real-world stimuli.

### Asymmetric Gate Modulation

Asymmetric design at the gate terminal offers another potent avenue for tailoring synaptic functionalities in FETs. Fundamentally, introducing geometrical or functional asymmetry to the gate structure such as employing half-gates or non-uniform gate dielectrics creates a lateral or graded electric field within the channel [119–121]. This inhomogeneous field profile directly modulates the spatial distribution of charge carriers, enabling precise and dynamic control over the channel conductance, which is the fundamental analogue to synaptic weight. When combined with 2D materials as the channel, this strategy gains further prominence. The atomically thin body of 2D semiconductors renders their electronic properties exceptionally sensitive to local gate fields, amplifying the impact of asymmetric gate designs. Furthermore, the pristine interfaces and mechanical flexibility of 2D materials allow for the innovative integration of heterogeneous or patterned gate stacks, paving the way for novel device architectures to achieve advanced biomimetic sensing, learning, and adaptation in neuromorphic systems. Here, we highlighted two design schemes of asymmetric gate, namely local gate structure and double gate structure.

**Fig. 10:**
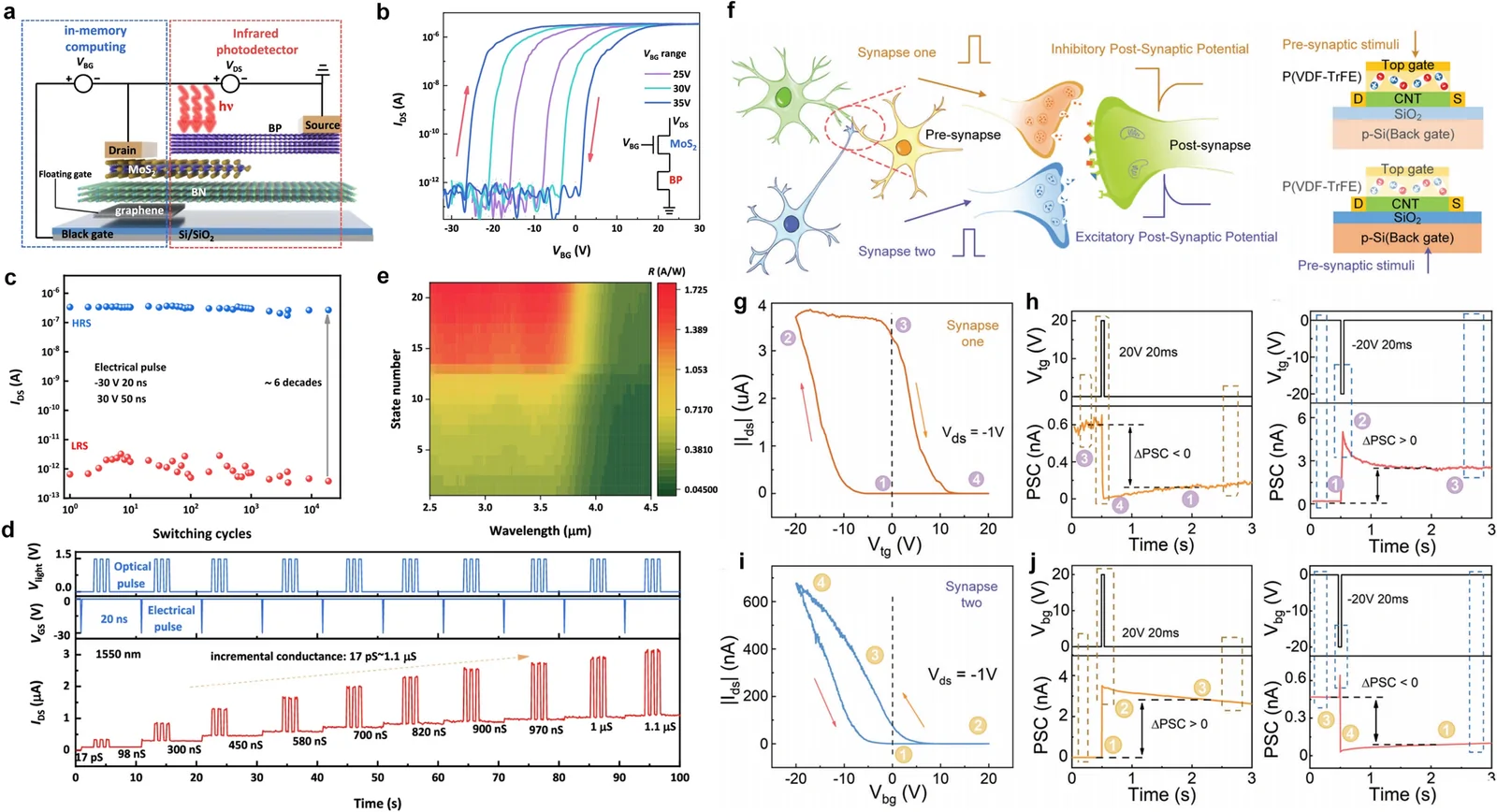
Neuromorphic FETs with asymmetric gate modulation. **a** Device structure of MoS_2_/BP heterojunction-based transistor with graphene semi-floating structure. **b** Hysteresis behaviors of the device under different bottom-gate voltage (*V*_*BG*_). **c** Cyclic change of conductance states by applying different electrical voltage pulses. **d** Real-time test for conductance configuration and the photo-response at different conductance states. **e** Responsivity mapping plots at different wavelengths for different conductivity states [122]. Copyright 2024, Springer Nature. **f** Schematic diagram of signal transmission between biological neurons and the corresponding operation mode in dual-gate synaptic FETs. **g** Hysteresis loop obtained in top-gating mode. **h** PSC change under positive and negative top-gate voltage spikes. **i** Hysteresis loop obtained in bottom-gating mode. **j** PSC change under positive and negative bottom-gate voltage spikes [106]. Copyright 2023, WILEY–VCH

Zhou et al. designed and fabricated a semi-floating-gate structure, where near- to mid-infrared photodetection, memory and computing (PMC) functionalities were integrated in a single device as shown in Fig. 10a [122]. Here, MoS_2_/BP heterojunction was chosen as conducting channel, h-BN worked as a tunneling layer underneath MoS_2_ channel and graphene was selected as a floating-gate layer to gate the MoS_2_ channel only, referring to a semi-floating-gate structure. Such semi-floating-gate region effectively modulated the conduction states and threshold voltage by controlling the carrier tunneling process between MoS_2_ and graphene (Fig. 10b). This mechanism enabled the structure to function as a memory element capable of achieving a wide range of conductance modulation (Fig. 10c). The change in the conductance state of MoS_2_ directly influenced the energy band alignment at the MoS_2_/BP heterojunction, thereby altering its photoresponse. This characteristic established a direct link between the memory state and optoelectronic performance. As shown in Fig. 10d, the photoresponse gradually enhanced with an increase in the conductance state. This correlation is further confirmed in Fig. 10e, which illustrated the relationship between responsivity, conductance state, and incident wavelength, demonstrating that a higher conductance state led to a stronger photoresponse across a broad spectral range. This unique feature was further validated through simulation tasks involving the processing and recognition of mid‑wave infrared images. Besides, integrating different gate dielectrics into a single device also introduced the asymmetric gate modulation as shown in Fig. 10f, where the top-gate dielectric was composed of organic ferroelectrics but the bottom-gate dielectric was the SiO_2_ layer functionalized by interface defects [106]. Befitting from the different memory mechanisms induced by ferroelectric polarization and charge trapping/detrapping, a bidirectional synaptic plasticity modulation was achieved. From Fig. 10g, the direction hysteresis loop was determined by ferroelectric polarization switching that contributed to an inhibitory PSC under positive voltage spikes but excitatory PSC under negative ones (Fig. 10h). Interestingly, the direction hysteresis loop was completely opposite observed in bottom-gating mode, which was dominated by charge-trapping/detrapping process (Fig. 10i, j). The bidirectional plasticity dynamics induced by the asymmetric gate dielectrics contributed to the controllable conversion between inhibitory and excitatory plasticity, which could be used to emulate the in self-adaptive characteristics of neural systems. The results above indicate that the asymmetric gate modulation is of significant importance for achieving reconfigurable plasticity and its dynamic modulation, which benefits from the structural complexity compared to other device designs.

## Summary and Perspectives

In summary, this review has systematically examined the deliberate incorporation of asymmetry across material, structural, and device levels as a powerful design principle for 2D neuromorphic devices. The core rationale lies in harnessing the intrinsic or engineered asymmetric physical properties such as heterogeneous ion migration, Schottky barrier modulation, built-in electric fields, and interfacial dipole formations to emulate and control the essential synaptic plasticity found in biological neural systems. These asymmetrically enabled mechanisms provide a robust physical foundation for implementing key neuromorphic functionalities, including STP/LTP, STDP, and reconfigurable plasticity, thereby bridging the gap between fundamental device physics and complex neuronal behaviors. Table 2 summarizes the asymmetric designs highlighted in this review and indicated the underlying relationship between them and neuromorphic functions. It should be noted that in this table, we primarily highlight the key physical characteristics of each design, which does not imply that each design possesses only that specific characteristic. In fact, the listed physical properties may be realized across multiple designs. Additionally, the neuromorphic functionalities presented are potential and prospective; namely, some have already been achieved through these designs, while others represent future applications derived from our perspectives on the corresponding physical properties. Overall, this table aims to provide researchers with inspiration in the field of neuromorphic device research. The field of 2D neuromorphic devices remains at a nascent stage, and a unified set of benchmarking protocols for evaluating device performance has yet to be established. This lack of standardization makes direct comparison across different device architectures challenging, as reported metrics often depend heavily on specific measurement conditions and device configurations. To provide a preliminary comparative assessment, we summarize the key neuromorphic metrics for representative asymmetric 2D devices alongside conventional counterparts in Table 3. Notably, asymmetric 2D devices demonstrate compelling strengths in several key metrics; for example, asymmetric contact engineering enables self-powered operation with ultra-low energy consumption down to 90 zJ per spike, representing a fundamental advantage for edge computing applications where power budget is severely constrained. Meanwhile, 2D Janus WSSe and ferroelectric CIPS devices exhibit excellent endurance exceeding 10^5^ and 10^4^ cycles, respectively, demonstrating reliable switching characteristics. These examples illustrate that asymmetric design strategies not only enable novel functionalities but also deliver competitive performance metrics that position them as promising candidates for next-generation neuromorphic hardware. However, it should be noted that the variations in reported performance metrics may not solely originate from the devices themselves but are also strongly influenced by differences in measurement protocols, device geometries, and testing environments. For instance, energy per spike values is often calculated under different pulse conditions, and dynamic range measurements may employ varying definitions of conductance states. This observation underscores the urgent need for the development of standardized testing methodologies and benchmark metrics tailored to neuromorphic devices, which would enable fair comparisons across studies and accelerate progress toward practical applications.

**Table 2:** The summary of the reviewed asymmetric designs and the underlying relationships between their physical features and potential neuromorphic functions

Level	Design paradigm	Typical example	Key Physical features	Potential Neuromorphic applications	References
Material	Anisotropic crystal structure	BP	Direction-Dependent Charge Transport and light–matter interaction	Polarization-Sensitive Vision Sensing System	[123]
	Janus atomic composition	WSSe	Built-in Vertical Electric Dipole	Temporal synaptic dynamics with enhanced plasticity and energy efficiency	[124]
	Symmetry-broken induced polarization	CIPS	Non-Volatile and Switchable Polarization	Precise control of memory characteristics in neuromorphic and in-memory computing devices	[125]
Structure	MDHs	2D/0D 2D/1D 2D/3D	Dimensionally confined carrier charge transports	Enhanced Learning Rules and Implementation of Spatiotemporal Computing	[126]
	Band alignment engineering	Straddling/Staggered/Broken Gap	Spatiotemporally modulated carrier dynamics	Multi-timescale synaptic plasticity and complex signal processing for associative learning	[86]
	Selective interface modification	Localized selective irradiation	Interface-controlled carrier dynamics	Precise tuning of synaptic behaviors and multi-state memory to emulate neural adaptability	[127]
Device	Asymmetric diffusive memristor	Al/MoS_2_/Poly-Si	Gradient-tunable memconductance states	Analog synaptic plasticity and support multi-level learning and memory	[128]
	Asymmetric contact engineering	Pd/MoS_2_/Ni/Au	Inherent differentiated Schottky barrier heights	Self-powered neuromorphic devices and systems with extremely low energy consumption	[12]
	Asymmetric gate modulation	Half-floating-gate transistor	Controllable spatial distribution of charge carriers	Reconfigurable neuromorphic devices endowed with multiple plasticity modulation strategies	[129]

**Table 3:** Comparison of key neuromorphic metrics between asymmetric 2D neuromorphic devices and traditional devices

Category	Typical design	Key neuromorphic metrics				References
		Weight update nonlinearity or linearity (*) (LTP/LTD)	Dynamic range	Endurance	Energy per spike	
Asymmetric 2D devices	Anisotropic ReSe_2_	–	27.5%	–	–	[11]
	2D Janus WSSe	5.65/2.4*	–	10^5^	119.5 pJ	[124]
	Ferroelectric CIPS	0.7/4.9	24.2	> 10^4^	–	[61]
	WSe_2_/InAs 2D/0D MDHs	0.0005/ − 0.002	1.2 (LTP) 1.81 (LTD)	–	33.5 pJ	[75]
	Type III Band alignment BP/SnS_2_	1.1–2.7	–	–	83 pJ	[88]
	Adsorbate-induced asymmetry	0.6 only for LTP	> 200	4000	*P*_prog_ < 100 pW, *P*_read_ < 1 pW	[99]
	Asymmetric diffusive memristor	2.6/ − 0.5	–	800	37 pJ	[130]
	Asymmetric Contact	–	–	–	90 zJ	[118]
	Asymmetric Gating	− 0.08/2.86	15.1	–	0.86 pJ	[106]
Traditional Symmetric Devices	COMS Transistor	0.06/0.21	> 10^3^	> 10^7^	415 pJ/m	[131]
	Oxide-based RRAM	–	2.5	> 200 k	0.51 fJ	[132]
	GeTe/Sb_2_Te_3_PCM	0.32/0.32	13.1	–	–	[133]

Strategic implementation of asymmetry has emerged as a powerful paradigm for enriching the functionality of 2D neuromorphic devices. However, translating this potential into practical technologies requires a balanced assessment of both opportunities and inherent challenges. This perspective critically examines the road ahead, addressing manufacturing trade-offs, system-level integration requirements, architectural opportunities, and the concrete steps needed to bridge the gap between laboratory demonstrations and functional neuromorphic hardware.

### Toward Manufacturability and Reliability: The Trade-offs of Asymmetric Design

While the techniques employed to introduce asymmetry enhance individual device performance, they are also well-established sources of increased device-to-device and cycle-to-cycle variability. At the material level, asymmetric designs rely on precisely controlled anisotropy, polarization, or stoichiometry, where minor variations during synthesis or transfer can alter the intended electronic properties [99, 134]. At the structural level, heterojunctions and engineered interfaces depend exquisitely on interfacial quality and alignment accuracy, meaning that nanometer-scale variations in layer overlap or contact configuration translate directly into large fluctuations in carrier transport [135]. At the device level, asymmetric configurations operate on the principle of controlled spatial imbalance, indicating that any non-uniformity is amplified rather than averaged out because device function hinges on the difference between intentionally mismatched regions [136]. This presents a critical challenge for wafer-scale integration, as maintaining process uniformity across large arrays becomes intrinsically more difficult when intentional asymmetry is introduced.

Current literature rarely reports device yield statistics for asymmetric 2D devices, yet this metric will be decisive for technological viability. This scarcity reflects the inherent limitations of current research in the field of 2D material-based neuromorphic devices because most studies remain at the proof-of-concept stage, focusing on individual device performance optimization rather than large-scale array integration [137]. The absence of wafer-scale fabrication demonstrations and systematic yield analysis underscores the gap between laboratory innovation and technological readiness. Despite these challenges, the pursuit of asymmetric 2D neuromorphic devices remains a critical research frontier, as the potential gains in functionality, energy efficiency, and bio-realistic computing far outweigh the obstacles. Mitigating variability while preserving desired asymmetric function will require co-optimization across multiple levels from material design through device engineering to circuit/algorithm-level solutions.

**Fig. 11:**
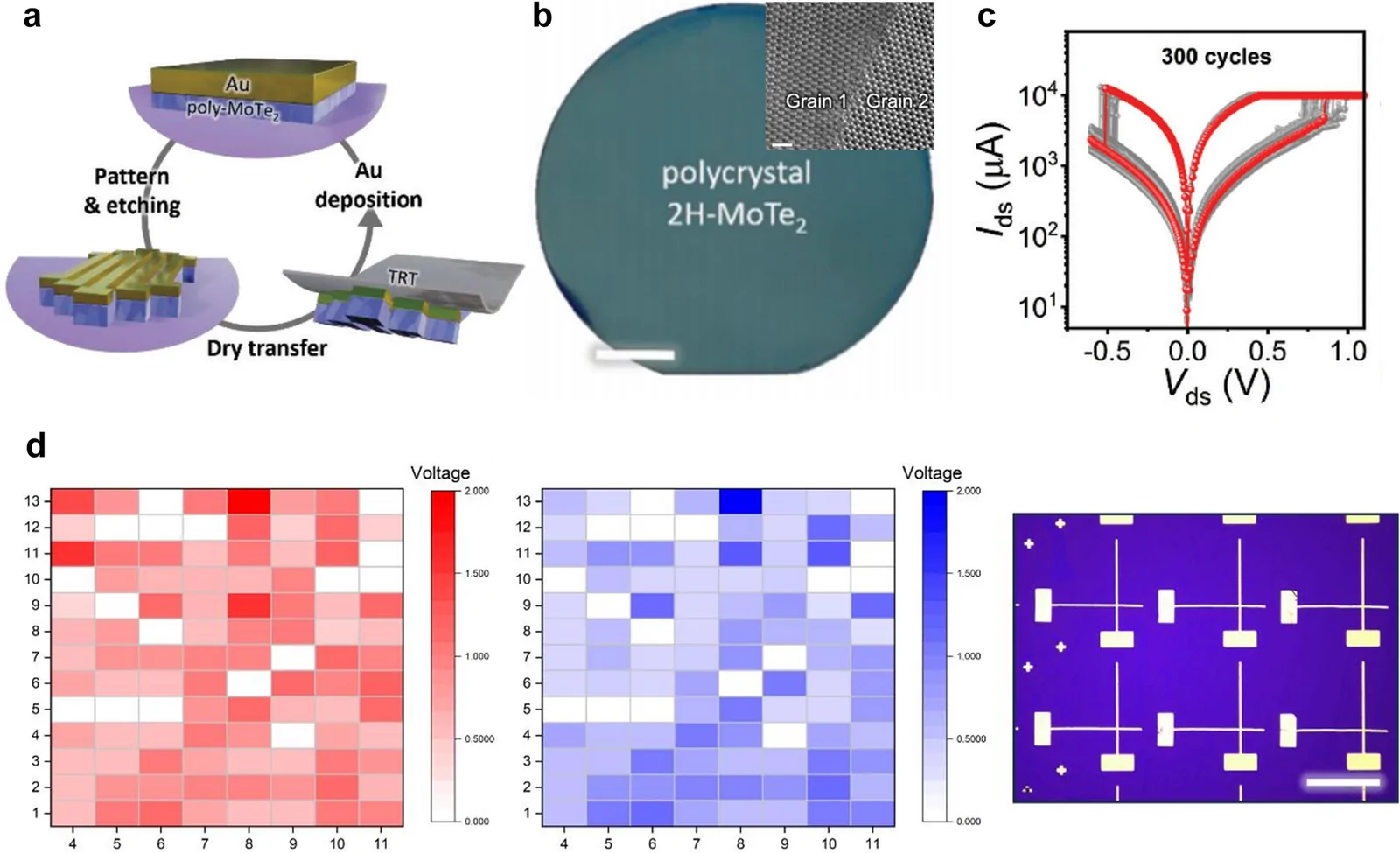
**a** Schematic of fabrication process for polycrystalline 2H-MoTe_2_ memristor crossbar array. **b** Photography image of the poly-MoTe_2_ film synthesized on a 2-inch wafer. Scale bar: 1.5 cm. The inset is atomic-resolution (AR-STEM) image of grain boundaries in the poly-MoTe_2_ film. Scale bar: 1 nm. **c** *I-V* curves for 300 cycles of the polycrystalline 2H-MoTe_2_ memristor. **d** Set voltage (red) and reset voltage (blue) measurement results for 104 memristors. Blanks indicate device failure. And the corresponding OM image of cross-point memristor array. Scale bar: 300 μm [138]. Copyright 2024, WILEY–VCH

Yang et al. provide a compelling example of this co-optimization strategy through wafer-scale fabrication of polycrystalline 2H-MoTe_2_ memristor arrays (Fig. 11a) [138]. Rather than attempting to eliminate asymmetry-induced variability, this work strategically engineers it by directly synthesizing highly polycrystalline 2H-MoTe_2_ films with high-density vertically aligned grain boundaries on 2-inch SiO_2_/Si wafers (Fig. 11b). These aligned GBs serve as confined and uniform diffusion pathways for metal ions, transforming stochastic defect formation into deterministic filament confinement. During resistive switching, Au ions migrate preferentially along the vertically aligned GBs rather than randomly disrupting the crystalline lattice. This design achieves reliable bipolar resistive switching with markedly reduced variability with cycle-to-cycle variation coefficients of 8.3% (Fig. 11c), device yields exceeding 83.6% across 104 devices (Fig. 11d). Critically, the approach preserves asymmetric function, where the GBs themselves constitute a structural asymmetry that directs ion migration, while mitigating its negative impact on uniformity. The work further demonstrates wafer-scale uniformity and a damage-free transfer process using pre-deposited metal electrodes, avoiding cracks and contamination typical of conventional PMMA-based transfer. This study illustrates that by engineering asymmetry itself and transforming randomly distributed intrinsic defects into ordered, predefined pathways, it is possible to retain the functional benefits of asymmetric design while overcoming the manufacturability challenges.

### Toward System Integration: From Single Device to Circuit-Level Integration

Expanding asymmetry from single devices to circuit and system level represents a critical frontier for neuromorphic computing. While individual asymmetric devices have demonstrated enhanced functionality, their true potential can only be realized through integration into functional circuits and architectures [139]. This transition is essential because practical neural computation requires not isolated synapses but interconnected networks capable of processing temporal information, performing parallel operations, and executing complex learning rules. System-level co-design, where network functionality directly leverages the physical properties of asymmetric building blocks such as directional signal filtering, hysteresis, or nonlinear response, offers a pathway beyond simple synapse emulation toward more efficient, brain-inspired computing systems.

**Fig. 12:**
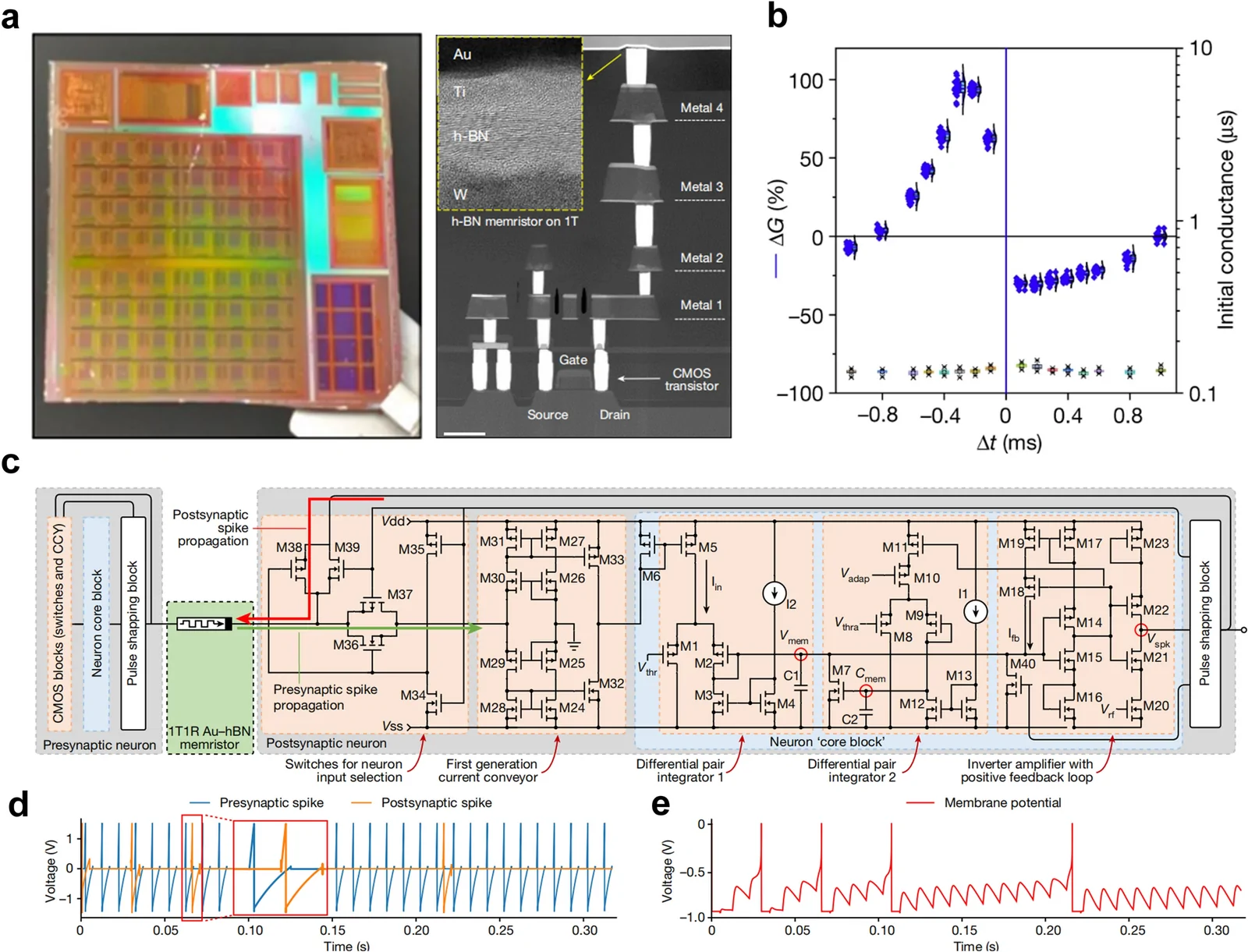
Hybrid 2D-CMOS microchips. **a** Photograph of the 2 cm × 2 cm microchips containing the CMOS circuitry and high-angle annular dark-field cross-sectional SEM image of a 1T1M cell in the crossbar array. Scale bar, 600 nm. **b** STDP characteristic of the 1T1M cell with Au-Ti-h-BN-W memristor. **c** Circuit schematic of the proposed neuron–synapse–neuron block combining h-BN-based 1T1M cells and CMOS circuitry. **d** SPICE simulation of the pre- and postsynaptic signals applied to the CMOS-h-BN-based 1T1M. **e** SPICE simulation of the neuron’s membrane potential [140]. Copyright 2023, The Author(s)

Current progress in this direction, while promising, remains at an early stage. Present investigations predominantly focus on using individual asymmetric devices as foundational elements for constructing basic neuromorphic circuits. Zhu et al. reported high-integration-density 2D-CMOS hybrid microchips, integrating h-BN memristors with CMOS transistors to form 1T1M units with asymmetric layered structures as shown in Fig. 12a [140]. This system-level co-design leverages asymmetric ion diffusion in memristors, enabled by CMOS control, realizing STDP characteristics essential for synapses (Fig. 12b). They further proposed a neuron–synapse–neuron circuit schematic (Fig. 12c) and validated pre-/postsynaptic signal transmission via SPICE simulation (Fig. 12d, e), achieving spiking neural networks with ~ 90% MNIST classification accuracy. This work represents a pivotal advance in circuit-level asymmetric neuromorphic systems, bridging device physics and practical computing. Another example is the work by Zhao et al. that strategically employed an asymmetric diffusive memristor as the core nonlinear and directional switching element to realize a compact one diffusive memristor, one transistor, and one resistor (1M1T1R) enabled spiking neuron circuit [141]. This design effectively transduced the inherent ionic dynamics and threshold characteristics of the asymmetric device into rich neuronal functionalities such as leaky integration, threshold firing, and a refractory period. This work illustrated a significant step in exploiting device-level asymmetry at the circuit level to create efficient and bio-inspired computational platform toward future complex neuromorphic system development.

Nonetheless, several challenges hinder the transition from individual devices to integrated circuit systems. The diversity of operating mechanisms across asymmetric designs complicates unified peripheral circuitry and programming protocols [142]. The absence of standardized compact models limits architecture-level simulations, and device variability induced by asymmetric design as discussed in 5.1 also degrades reliability as arrays scale [143]. Addressing these challenges requires coordinated efforts such as developing design frameworks that map neural network architectures onto device characteristics and implementing variability-aware techniques such as adaptive biasing.

### Toward In-Sensor Computing: Architectural Advantages and Challenges

The asymmetric design strategies discussed throughout this review offer distinct advantages for realizing integrated sensing-memory-computing architectures, a paradigm broadly termed in-sensor computing [144]. Conventional neuromorphic systems, inspired by foundational perspectives on in-sensor computing, typically rely on separate functional blocks for sensory transduction, analog-to-digital conversion, and neural network processing, leading to significant data movement overhead and energy consumption [145]. Asymmetric 2D neuromorphic devices provide a pathway to overcome these limitations by enabling the co-localization of multiple functionalities within a single device unit.

**Fig. 13:**
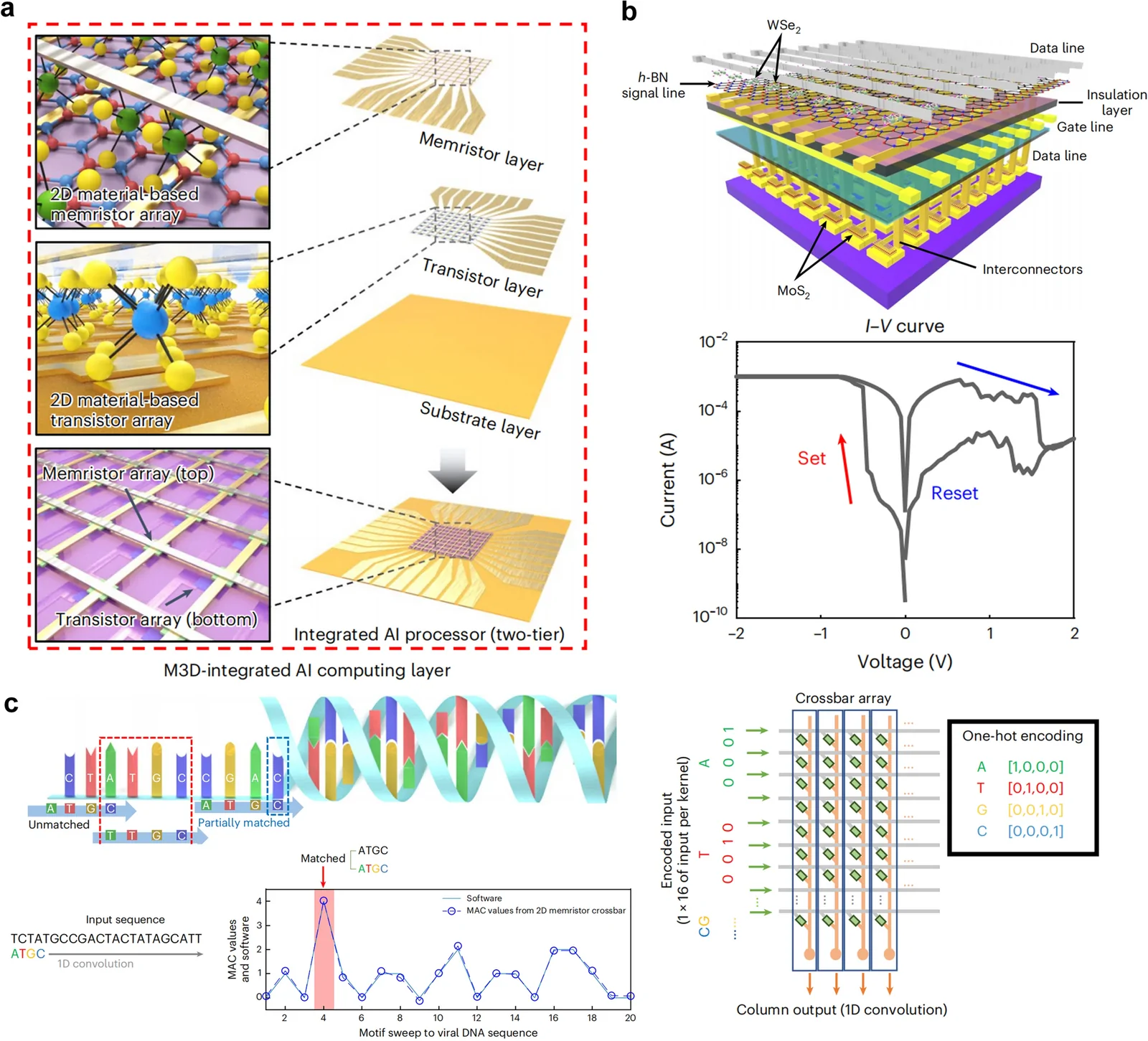
Monolithic 3D integration of 2D material-based AI processing hardware. **a** Schematic illustration of in-sensor computing system based on M3D-integrated, 2D material-based electronics. **b** Schematic diagram of M3D-integrated AI processor comprising WSe_2_/h-BN-based memristors and MoS_2_-based transistors (top) and its DC switching performance (bottom). **c** DNA motif discovery using this M3D-integrated, 2D material-based AI system [146]. Copyright 2023, The Author(s)

The architectural advantages of asymmetric 2D neuromorphic devices for in-sensor computing stem from three intrinsic characteristics. First, the inherent anisotropy and polarization effects in asymmetric material systems such as anisotropic 2D materials allow direct encoding of sensory information, including light intensity, wavelength, or polarization, into conductance states without separate sensor frontends [123]. Second, the nonlinear and hysteretic switching dynamics arising from asymmetric device configurations naturally implement synaptic weight update rules and temporal integration functions essential for neural computation [128]. Third, the atomic thickness and van der Waals integration capability of 2D materials enable monolithic three-dimensional (M3D) stacking of sensing, memory, and processing layers with minimal footprint and parasitic capacitance [146]. Kang et al. successfully stacked six layers of device arrays comprising MoS_2_ transistors and WSe_2_/h-BN memristors into a fully integrated AI processing system (Fig. 13a) [146]. This work illustrates how the WSe_2_/h-BN double-layer configuration as a device-level asymmetry enables reliable memristive switching with controlled ion migration pathways (Fig. 13b), while the 1T1M integration scheme as a circuit-level asymmetry allows precise programming of conductance states via gate-controlled current limiting. Such vertically integrated architecture achieves key in-sensor computing objectives, including dense interlayer connectivity that minimizes routing paths and parasitic capacitance, parallel processing across stacked layers that reduces latency and voltage drops, and mechanical flexibility for wearable integration. The demonstration of DNA motif discovery using this M3D-integrated system further validates how asymmetric device characteristics can be harnessed for practical computing tasks (Fig. 13c). This work provides concrete validation that asymmetric 2D devices can be organized into vertically integrated systems realizing co-localization of memory and processing with unprecedented density and efficiency, establishing a template for moving beyond incremental device optimization toward transformative system architectures.

Despite these promising advantages, significant challenges remain before asymmetric 2D devices can realize their full potential in integrated intelligent systems. The sensitivity to environmental perturbations that enables versatile sensory responses simultaneously introduces instability in memory retention and computing accuracy. Furthermore, the diversity of operating mechanisms across different asymmetric designs complicates the development of unified peripheral circuitry and programming protocols required for large-scale array integration. Addressing these challenges requires coordinated progress across multiple fronts such as developing encapsulation strategies that stabilize device operation without compromising sensory responsiveness [147], designing hybrid architectures that strategically allocate different asymmetric functions to optimized sub-blocks, and establishing standardized interfaces between 2D device layers and CMOS peripheral circuits [148].

Looking toward future technological translation, several asymmetry strategies emerge as particularly promising for large-scale integration based on their compatibility with wafer-scale control. Material-level approaches that embed asymmetry during synthesis, such as intrinsic anisotropy of 2D materials introduced by controllable growth, offer inherent scalability because asymmetry is defined by growth conditions rather than post-fabrication patterning. These strategies transform the challenge of variability control from one of suppressing random fluctuations into one of engineering deterministic, uniform structural features across entire wafers. Device-level strategies leveraging asymmetric contact engineering and gate modulation present more immediate integration pathways, as they build upon established microfabrication techniques and have already demonstrated compatibility with CMOS hybrid integration. The convergence of these strategies toward wafer-scale manufacturability, rather than isolated device optimization, will ultimately determine whether asymmetric 2D neuromorphic devices transition from laboratory curiosities to platform technologies for next-generation computing. By providing a direct pathway to encode multi-level conductance states and dynamic synaptic weights within structures amenable to large-scale fabrication, asymmetry serves as a cornerstone for translating the remarkable capabilities of biological neural networks into practical solid-state hardware. Continued exploration along these directions, with explicit attention to the manufacturability criteria outlined throughout this perspective, will be vital for unlocking the full potential of 2D materials in creating next-generation, low-power, and adaptive neuromorphic computing technologies.

## References

[1] S. Wang, M. Wu, W. Liu, J. Liu, Y. Tian et al., Dopamine detection and integration in neuromorphic devices for applications in artificial intelligence. Device **2**(2), 100284 (2024). 10.1016/j.device.2024.100284

[2] J. Yao, Y. Teng, Q. Wang, Y. He, L. Liu et al., Advancing intelligent neuromorphic computing: recent progress in all-optical-controlled artificial synaptic devices. ACS Nano **19**(29), 26320–26346 (2025). 10.1021/acsnano.5c05240 40679440 10.1021/acsnano.5c05240

[3] M.H. Pervez, E. Elahi, M.A. Khan, M. Nasim, M. Asim et al., Recent developments on novel 2D materials for emerging neuromorphic computing devices. Small Struct. **6**(2), 2400386 (2025). 10.1002/sstr.202400386

[4] V. Milo, G. Malavena, C. Monzio Compagnoni, D. Ielmini, Memristive and CMOS devices for neuromorphic computing. Materials **13**(1), 166 (2020). 10.3390/ma13010166 31906325 10.3390/ma13010166 PMC6981548

[5] Y. Sun, Y. Ding, D. Xie, Mixed-dimensional van der Waals heterostructures enabled optoelectronic synaptic devices for neuromorphic applications. Adv. Funct. Mater. **31**(47), 2105625 (2021). 10.1002/adfm.202105625

[6] Q. He, H. Wang, Y. Zhang, A. Chen, Y. Fu et al., Two-dimensional materials based two-transistor-two-resistor synaptic kernel for efficient neuromorphic computing. Nat. Commun. **16**, 4340 (2025). 10.1038/s41467-025-59815-x 40346103 10.1038/s41467-025-59815-x PMC12064777

[7] T.C. Südhof, Neurotransmitter release: the last millisecond in the life of a synaptic vesicle. Neuron **80**(3), 675–690 (2013). 10.1016/j.neuron.2013.10.022 24183019 10.1016/j.neuron.2013.10.022 PMC3866025

[8] R.A. Nicoll, A brief history of long-term potentiation. Neuron **93**(2), 281–290 (2017). 10.1016/j.neuron.2016.12.015 28103477 10.1016/j.neuron.2016.12.015

[9] X. Li, H. Liu, C. Ke, W. Tang, M. Liu et al., Review of anisotropic 2D materials: controlled growth, optical anisotropy modulation, and photonic applications. Laser Photonics Rev. **15**(12), 2100322 (2021). 10.1002/lpor.202100322

[10] Z. Yang, Z. Yang, L. Liu, X. Li, J. Li et al., Anisotropic mass transport enables distinct synaptic behaviors on 2D material surface. Mater. Today Electron. **5**, 100047 (2023). 10.1016/j.mtelec.2023.100047

[11] Y. Zhu, Y. Tao, Z. Wang, J. Bian, Z. Li et al., In-plane anisotropic two-dimensional ReSe_2_ optoelectronic memristor for a polarization-sensitive neuromorphic vision system. ACS Nano **19**(27), 25480–25489 (2025). 10.1021/acsnano.5c08221 40577553 10.1021/acsnano.5c08221

[12] R.K. Pandey, S. Baek, N. Lee, S.-M. Lee, S. Lee, Asymmetric contact van der Waals ferroelectric transistors for self-powered multifunctional artificial visual system. ACS Nano **19**(39), 35071–35080 (2025). 10.1021/acsnano.5c12376 40994192 10.1021/acsnano.5c12376

[13] A.N. Rudenko, M.I. Katsnelson, Anisotropic effects in two-dimensional materials. 2D Mater. **11**(4), 042002 (2024). 10.1088/2053-1583/ad64e1

[14] W. Ahmad, Y. Wang, J. Kazmi, U. Younis, N.M. Mubarak et al., Janus 2D transition metal dichalcogenides: research progress, optical mechanism and future prospects for optoelectronic devices. Laser Photonics Rev. **19**(6), 2400341 (2025). 10.1002/lpor.202400341

[15] Y.-F. Zhang, H. Guo, Y. Zhu, S. Song, X. Zhang et al., Emerging multifunctionality in 2D ferroelectrics: a theoretical review of the interplay with magnetics, valleytronics, mechanics, and optics. Adv. Funct. Mater. **34**(51), 2410240 (2024). 10.1002/adfm.202410240

[16] C. Chen, Y. Zhou, L. Tong, Y. Pang, J. Xu, Emerging 2D ferroelectric devices for in-sensor and in-memory computing. Adv. Mater. **37**(2), 2400332 (2025). 10.1002/adma.202400332 38739927 10.1002/adma.202400332 PMC11733831

[17] H. Wang, Z. Liu, Y. Sun, X. Ping, J. Xu et al., Anisotropic electrical properties of aligned PtSe_2_ nanoribbon arrays grown by a pre-patterned selective selenization process. Nano Res. **15**(5), 4668–4676 (2022). 10.1007/s12274-022-4110-3

[18] C. Wang, G. Zhang, S. Huang, Y. Xie, H. Yan, The optical properties and plasmonics of anisotropic 2D materials. Adv. Opt. Mater. **8**(5), 1900996 (2020). 10.1002/adom.201900996

[19] J. Yang, C. Liu, H. Xie, W. Yu, Anisotropic heat transfer properties of two-dimensional materials. Nanotechnology **32**(16), 162001 (2021). 10.1088/1361-6528/abdb15 33434892 10.1088/1361-6528/abdb15

[20] Z.-D. Gao, Z.-H.-Y. Jiang, J.-D. Li, B.-W. Li, Y.-Y. Long et al., Anisotropic mechanics of 2D materials. Adv. Eng. Mater. **24**(11), 2200519 (2022). 10.1002/adem.202200519

[21] J. He, D. He, Y. Wang, Q. Cui, M.Z. Bellus et al., Exceptional and anisotropic transport properties of photocarriers in black phosphorus. ACS Nano **9**(6), 6436–6442 (2015). 10.1021/acsnano.5b02104 25961945 10.1021/acsnano.5b02104

[22] P.K. Venuthurumilli, P.D. Ye, X. Xu, Plasmonic resonance enhanced polarization-sensitive photodetection by black phosphorus in near infrared. ACS Nano **12**(5), 4861–4867 (2018). 10.1021/acsnano.8b01660 29684270 10.1021/acsnano.8b01660

[23] Z. Luo, J. Maassen, Y. Deng, Y. Du, R.P. Garrelts et al., Anisotropic in-plane thermal conductivity observed in few-layer black phosphorus. Nat. Commun. **6**, 8572 (2015). 10.1038/ncomms9572 26472191 10.1038/ncomms9572 PMC4634212

[24] J. Tao, W. Shen, S. Wu, L. Liu, Z. Feng et al., Mechanical and electrical anisotropy of few-layer black phosphorus. ACS Nano **9**(11), 11362–11370 (2015). 10.1021/acsnano.5b05151 26422521 10.1021/acsnano.5b05151

[25] H. Liu, C. Zhu, Y. Chen, X. Yi, X. Sun et al., Polarization-sensitive photodetectors based on highly in-plane anisotropic violet phosphorus with large dichroic ratio. Adv. Funct. Mater. **34**(17), 2314838 (2024). 10.1002/adfm.202314838

[26] D. Lu, J. Tan, M. Zhang, M. Shi, X. Feng et al., Probing the temperature-dependent thermal conductivity of violet phosphorus *via* optothermal Raman spectroscopy. J. Phys. Chem. Lett. **15**(35), 8942–8948 (2024). 10.1021/acs.jpclett.4c02000 39177269 10.1021/acs.jpclett.4c02000

[27] J. Singh, M. Jakhar, A. Kumar, K. Tankeshwar, Anisotropic and high carrier mobility of 2D α-te. Dae Solid State Physics Symposium 2019 Jodhpur, India. AIP Publishing, (2020): 030693. 10.1063/5.0017367

[28] J. Zhang, J. Liu, Y. Tian, J. Guo, W. Kong et al., Polarization-sensitive and wide-spectrum photodetector from ultraviolet to near-infrared light based on 2D tellurium at room temperature. Appl. Surf. Sci. **670**, 160685 (2024). 10.1016/j.apsusc.2024.160685

[29] S. Huang, M. Segovia, X. Yang, Y.R. Koh, Y. Wang et al., Anisotropic thermal conductivity in 2D tellurium. 2D Mater. **7**(1), 015008 (2020). 10.1088/2053-1583/ab4eee

[30] H. Ma, W. Hu, J. Yang, Control of highly anisotropic electrical conductance of tellurene by strain-engineering. Nanoscale **11**(45), 21775–21781 (2019). 10.1039/c9nr05660b 31701993 10.1039/c9nr05660b

[31] F. Chu, M. Chen, Y. Wang, Y. Xie, B. Liu et al., A highly polarization sensitive antimonene photodetector with a broadband photoresponse and strong anisotropy. J. Mater. Chem. C **6**(10), 2509–2514 (2018). 10.1039/C7TC05488B

[32] G. Liu, H. Wang, G.-L. Li, D. Wang, Giant anisotropy of thermal expansion and thermomechanical properties of monolayer α-antimonene: A first-principles study. Comput. Mater. Sci. **169**, 109132 (2019). 10.1016/j.commatsci.2019.109132

[33] L. Pi, C. Hu, W. Shen, L. Li, P. Luo et al., Highly in-plane anisotropic 2D PdSe_2_ for polarized photodetection with orientation selectivity. Adv. Funct. Mater. **31**(3), 2006774 (2021). 10.1002/adfm.202006774

[34] L. Chen, W. Zhang, H. Zhang, J. Chen, C. Tan et al., In-plane anisotropic thermal conductivity of low-symmetry PdSe_2_. Sustainability **13**(8), 4155 (2021). 10.3390/su13084155

[35] C. Long, Y. Liang, H. Jin, B. Huang, Y. Dai, PdSe2: flexible two-dimensional transition metal dichalcogenides monolayer for water splitting photocatalyst with extremely low recombination rate. ACS Appl. Energy Mater. **2**(1), 513–520 (2019). 10.1021/acsaem.8b01521

[36] R. Wang, X. Xu, Y. Yu, M. Ran, Q. Zhang et al., The mechanism of the modulation of electronic anisotropy in two-dimensional ReS_2_. Nanoscale **12**(16), 8915–8921 (2020). 10.1039/D0NR00518E 32266914 10.1039/d0nr00518e

[37] F. Liu, S. Zheng, X. He, A. Chaturvedi, J. He et al., Highly sensitive detection of polarized light using anisotropic 2D ReS_2_. Adv. Funct. Mater. **26**(8), 1169–1177 (2016). 10.1002/adfm.201504546

[38] Y.-D. Cao, Y.-H. Sun, S.-F. Shi, R.-M. Wang, Anisotropy of two-dimensional ReS2 and advances in its device application. Rare Met. **40**(12), 3357–3374 (2021). 10.1007/s12598-021-01781-6

[39] W.-L. Tao, Y.-Q. Zhao, Z.-Y. Zeng, X.-R. Chen, H.-Y. Geng, Anisotropic thermoelectric materials: pentagonal PtM_2_ (M = S, Se, Te). ACS Appl. Mater. Interfaces **13**(7), 8700–8709 (2021). 10.1021/acsami.0c19460 33556242 10.1021/acsami.0c19460

[40] J. Guo, Y. Liu, Y. Ma, E. Zhu, S. Lee et al., Few-layer GeAs field-effect transistors and infrared photodetectors. Adv. Mater. **30**(21), e1705934 (2018). 10.1002/adma.201705934 29611222 10.1002/adma.201705934

[41] Z. Zhou, M. Long, L. Pan, X. Wang, M. Zhong et al., Perpendicular optical reversal of the linear dichroism and polarized photodetection in 2D GeAs. ACS Nano **12**(12), 12416–12423 (2018). 10.1021/acsnano.8b06629 30408410 10.1021/acsnano.8b06629

[42] X. Jiang, T. Zhao, D. Wang, Anisotropic ductility and thermoelectricity of van der Waals GeAs. Phys. Chem. Chem. Phys. **25**(40), 27542–27552 (2023). 10.1039/d3cp03119e 37801049 10.1039/d3cp03119e

[43] R. Lu, Y. Li, H. Song, J. Jiang, Recent advances in emerging polarization-sensitive materials: from linear/circular polarization detection to neuromorphic device applications. Adv. Funct. Mater. **35**(24), 2423770 (2025). 10.1002/adfm.202423770

[44] R. Khan, N.U. Rehman, S. Kalluri, S. Elumalai, A. Saritha et al., 2D MoTe_2_ memristors for energy-efficient artificial synapses and neuromorphic applications. Nanoscale **17**(21), 13174–13206 (2025). 10.1039/d5nr01509j 40370074 10.1039/d5nr01509j

[45] D. Xie, K. Yin, Z.-J. Yang, H. Huang, X. Li et al., Polarization-perceptual anisotropic two-dimensional ReS_2_ neuro-transistor with reconfigurable neuromorphic vision. Mater. Horiz. **9**(5), 1448–1459 (2022). 10.1039/d1mh02036f 35234765 10.1039/d1mh02036f

[46] G. Peng, C. Zhang, M. Li, J. Zhang, C. Wu et al., Polarization-sensitive photodetectors based on anisotropic 2D selenium and its multifunctional applications. ACS Appl. Mater. Interfaces **17**(39), 55074–55083 (2025). 10.1021/acsami.5c14109 40970905 10.1021/acsami.5c14109

[47] X. Zheng, Y. Zhou, Y. Guo, Symmetry manipulation of two-dimensional semiconductors by Janus structure. Acc. Mater. Res. **6**(2), 124–128 (2025). 10.1021/accountsmr.4c00236

[48] R. Chaurasiya, S. Tyagi, A.J. Kale, G.K. Gupta, R. Kumar et al., Advances in physics and chemistry of transition metal dichalcogenide Janus monolayers: properties, applications, and future prospects. Adv. Theory Simul. **8**(4), 2400854 (2025). 10.1002/adts.202400854

[49] R. Jana, S. Ghosh, R. Bhunia, A. Chowdhury, Recent developments in the state-of-the-art optoelectronic synaptic devices based on 2D materials: a review. J. Mater. Chem. C **12**(15), 5299–5338 (2024). 10.1039/D4TC00371C

[50] J. Meng, T. Wang, H. Zhu, L. Ji, W. Bao et al., Integrated in-sensor computing optoelectronic device for environment-adaptable artificial retina perception application. Nano Lett. **22**(1), 81–89 (2022). 10.1021/acs.nanolett.1c03240 34962129 10.1021/acs.nanolett.1c03240

[51] Y. Wang, B. Han, M. Mayor, P. Samorì, Opto-electrochemical synaptic memory in supramolecularly engineered Janus 2D MoS_2_. Adv. Mater. **36**(8), e2307359 (2024). 10.1002/adma.202307359 37903551 10.1002/adma.202307359

[52] H. Zhu, T. Li, L. Fu, J. Bai, S. Li et al., A proprioceptive Janus fiber with controllable multistage segments for bionic soft robots. ACS Nano **18**(46), 32023–32037 (2024). 10.1021/acsnano.4c10117 39499810 10.1021/acsnano.4c10117

[53] X. Wang, Y. Sun, F. Liu, R. Zhang, T. Wang et al., Hierarchically engineered Janus fiber-hydrogel architecture: an ultrathin biomimetic skin for multiple-response sensing. Chem. Eng. J. **522**, 168206 (2025). 10.1016/j.cej.2025.168206

[54] C. Wang, L. You, D. Cobden, J. Wang, Towards two-dimensional van der Waals ferroelectrics. Nat. Mater. **22**(5), 542–552 (2023). 10.1038/s41563-022-01422-y 36690757 10.1038/s41563-022-01422-y

[55] K. Yang, H. Wan, J. Yu, H. Fu, J. Zhang et al., Interfacial polarization enhanced ultrafast carrier dynamics in ferroelectric CuInP_2_S_6_. Nano Lett. **25**(5), 1890–1897 (2025). 10.1021/acs.nanolett.4c05369 39905943 10.1021/acs.nanolett.4c05369

[56] Q. He, Z. Tang, M. Dai, H. Shan, H. Yang et al., Epitaxial growth of large area two-dimensional ferroelectric α-In_2_Se_3_. Nano Lett. **23**(7), 3098–3105 (2023). 10.1021/acs.nanolett.2c04289 36779554 10.1021/acs.nanolett.2c04289

[57] Y. Liu, W. Tang, J. Zeng, C. Bai, K. Zhou et al., Ferroelectric-based neuromorphic memory devices for bio-inspired computing. Nat. Rev. Electr. Eng. **2**(11), 773–787 (2025). 10.1038/s44287-025-00222-1

[58] S. Baek, Y.K. Kim, S.-M. Lee, H. Choi, J.-S. Park et al., Steep-slope CuInP2S6 ferroionic threshold switching field-effect transistor for implementation of artificial spiking neuron. Adv. Mater. **37**(44), e06921 (2025). 10.1002/adma.202506921 40817593 10.1002/adma.202506921 PMC12592917

[59] Z. Wang, F. Li, Y. Zhao, Z. Wang, Y. Zhang et al., Artificial synapses based on CIPS/Te vdW heterojunction ferroelectric transistor for traffic light recognition. Appl. Phys. Lett. **126**(21), 213501 (2025). 10.1063/5.0256495

[60] M. Li, Y. He, C. Wang, W.F. Io, F. Guo et al., Memristors based on ferroelectric Cu-deficient copper indium thiophosphate for multilevel storage and neuromorphic computing. Small 2412314 (2025). 10.1002/smll.202412314 10.1002/smll.202412314 40545880

[61] J. Niu, J. Lyu, J. Li, K.S. Samantaray, C. Jang et al., All-optical control of bidirectional polarization switching in ferroelectric heterostructures for neuromorphic and in-memory computing. Adv. Sci. (2026). 10.1002/advs.202522092 10.1002/advs.202522092 PMC13067862 41608983

[62] S.-J. Kang, W. Jung, O.H. Gwon, H.S. Kim, H.R. Byun et al., Photo-assisted ferroelectric domain control for α-In_2_Se_3_ artificial synapses inspired by spontaneous internal electric fields. Small **20**(22), 2470174 (2024). 10.1002/smll.202470174 10.1002/smll.202307346 38213011

[63] J. Zhou, A. Chen, Y. Zhang, D. Pu, B. Qiao et al., 2D ferroionics: conductive switching mechanisms and transition boundaries in van der Waals layered material CuInP_2_S_6_. Adv. Mater. **35**(38), 2302419 (2023). 10.1002/adma.202302419 10.1002/adma.202302419 37352331

[64] L. You, F. Liu, H. Li, Y. Hu, S. Zhou et al., In-plane ferroelectricity in thin flakes of van der Waals hybrid perovskite. Adv. Mater. **30**(51), 1803249 (2018). 10.1002/adma.201803249 10.1002/adma.201803249 30334281

[65] X. Wang, K. Yasuda, Y. Zhang, S. Liu, K. Watanabe et al., Interfacial ferroelectricity in rhombohedral-stacked bilayer transition metal dichalcogenides. Nat. Nanotechnol. **17**(4), 367–371 (2022). 10.1038/s41565-021-01059-z 35039684 10.1038/s41565-021-01059-z

[66] S. Son, Y. Lee, J.H. Kim, B.H. Kim, C. Kim et al., Multiferroic-enabled magnetic-excitons in 2D quantum-entangled van der Waals antiferromagnet NiI_2_. Adv. Mater. **34**(10), 2109144 (2022). 10.1002/adma.202109144 10.1002/adma.202109144 34936713

[67] R. Bian, C. Li, Q. Liu, G. Cao, Q. Fu et al., Recent progress in the synthesis of novel two-dimensional van der Waals materials. Natl. Sci. Rev. **9**(5), nwab164 (2021). 10.1093/nsr/nwab164 35591919 10.1093/nsr/nwab164 PMC9113016

[68] J. Xu, H. Che, C. Tang, B. Liu, Y. Ao, Tandem fields facilitating directional carrier migration in van der Waals heterojunction for efficient overall piezo-synthesis of H_2_O_2_. Adv. Mater. **36**(32), 2404539 (2024). 10.1002/adma.202404539 10.1002/adma.202404539 38810126

[69] F. Zhang, H. Shi, Y. Yu, S. Liu, D. Liu et al., Dynamic band-alignment modulation in MoTe_2_/SnSe_2_ heterostructure for high performance photodetector. Adv. Opt. Mater. **12**(16), 2303088 (2024). 10.1002/adom.202303088

[70] Z. Zhang, D. Yang, H. Li, C. Li, Z. Wang et al., 2D materials and van der Waals heterojunctions for neuromorphic computing. Neuromorph. Comput. Eng. **2**(3), 032004 (2022). 10.1088/2634-4386/ac8a6a

[71] D. Jariwala, T.J. Marks, M.C. Hersam, Mixed-dimensional van der Waals heterostructures. Nat. Mater. **16**(2), 170–181 (2017). 10.1038/nmat4703 27479211 10.1038/nmat4703

[72] T. Zhao, W. Yue, Q. Deng, W. Chen, C. Luo et al., Neuromorphic transistors integrating photo-sensor, optical memory and visual synapses for artificial vision application. Adv. Mater. **37**(27), 2419208 (2025). 10.1002/adma.202419208 10.1002/adma.202419208 40231687

[73] W. Fan, H. Yan, X. Wang, L. Tong, W. Yan et al., Polarization-sensitive photosynapse based on PdSe_2_/WS_2_ heterostructure for visible-infrared broadband artificial vision system. Adv. Funct. Mater. **35**(43), 2416703 (2025). 10.1002/adfm.202416703

[74] K. Liao, K. Ding, S. Li, X. Zhang, Y. Bi et al., Enhanced near-infrared photodetection in a mixed-dimensional 0D/2D heterostructure *via* two-photon absorption. Laser Photonics Rev. **19**(9), 2401352 (2025). 10.1002/lpor.202401352

[75] S. Shim, S. Kim, D. Lee, H. Kim, M.J. Kwon et al., Infrared-triggered retinomorphic artificial synapse electronic device containing multi-dimensional van der Waals heterojunctions. Small **21**(24), 2410892 (2025). 10.1002/smll.202410892 40033879 10.1002/smll.202410892 PMC12177863

[76] F. Ferrarese Lupi, G. Milano, A. Angelini, M. Rosero-Realpe, I. Murataj et al., Enhanced photoluminescence in a neuromorphic 2D memitter based on WS_2_*via* plasmonic nanoparticle self-assembly. ACS Appl. Mater. Interfaces **17**(24), 35695–35704 (2025). 10.1021/acsami.5c03059 40468870 10.1021/acsami.5c03059 PMC12186229

[77] L. Dong, B. Xue, G. Wei, S. Yuan, M. Chen et al., Highly promising 2D/1D BP-C/CNT bionic opto-olfactory co-sensory artificial synapses for multisensory integration. Adv. Sci. **11**(29), 2403665 (2024). 10.1002/advs.202403665 10.1002/advs.202403665 PMC11304314 38828870

[78] X. Li, K. Liu, D. Wu, P. Lin, Z. Shi et al., Van der Waals hybrid integration of 2D semimetals for broadband photodetection. Adv. Mater. **37**(48), 2415717 (2025). 10.1002/adma.202415717 39945105 10.1002/adma.202415717 PMC12676102

[79] H.-F. Li, J. Liu, S. Geng, T. Sun, Z. Lv et al., A 2D-3D perovskite memristor-based light-induced sensitized neuron for visual information processing. Adv. Mater. **37**(42), e08342 (2025). 10.1002/adma.202508342 40762277 10.1002/adma.202508342

[80] J. Kang, L. Zhang, S.-H. Wei, A unified understanding of the thickness-dependent bandgap transition in hexagonal two-dimensional semiconductors. J. Phys. Chem. Lett. **7**(4), 597–602 (2016). 10.1021/acs.jpclett.5b02687 26800573 10.1021/acs.jpclett.5b02687

[81] Y. Jin, K. Yu, A review of optics-based methods for thickness and surface characterization of two-dimensional materials. J. Phys. D Appl. Phys. **54**(39), 393001 (2021). 10.1088/1361-6463/ac0f1f

[82] Y. Mao, L. Wang, C. Chen, Z. Yang, J. Wang, Thickness determination of ultrathin 2D materials empowered by machine learning algorithms. Laser Photonics Rev. **17**(4), 2200357 (2023). 10.1002/lpor.202200357

[83] X. Sun, C. Zhu, X. Zhu, J. Yi, Y. Liu et al., Recent advances in two-dimensional heterostructures: from band alignment engineering to advanced optoelectronic applications. Adv. Electron. Mater. **7**(7), 2001174 (2021). 10.1002/aelm.202001174

[84] H. Xu, Y. Xue, Z. Liu, Q. Tang, T. Wang et al., Van der Waals heterostructures for photoelectric, memory, and neural network applications. Small Sci. **4**(4), 2470012 (2024). 10.1002/smsc.202470012 10.1002/smsc.202300213 PMC11935099 40212994

[85] X.-F. Luo, X.-B. Guo, D. Zhang, Q.-J. Sun, W.-H. Li et al., 2D Van der Waals heterostructure memristors: from band structure regulation to neuromorphic computing applications. Mater. Horiz. **12**(18), 7277–7304 (2025). 10.1039/d5mh00306g 40509874 10.1039/d5mh00306g

[86] X. He, X. Zhu, Z. Hong, B. Wang, W. Hong et al., Van der Waals heterojunction based self-powered biomimetic dual-mode sensor for precise object identification. Adv. Mater. **36**(49), 2411121 (2024). 10.1002/adma.202411121 10.1002/adma.202411121 39428861

[87] Y. Zhang, Y. Tang, K. Liu, Y. Gu, L. Wang et al., Optoelectronic synapse based on Te/SnS_2_ heterostructure with integrated sensing-memory-computing for neuromorphic visual system. Adv. Opt. Mater. **13**(26), e01371 (2025). 10.1002/adom.202501371

[88] W. Lv, Y. Zeng, X. Wang, W. Lv, X. Wu et al., Artificial synapse based on black phosphorus/SnS_2_ heterostructure transistor for neuromorphic computing with high accuracy. ACS Omega **10**(42), 49766–49775 (2025). 10.1021/acsomega.5c04929 41179224 10.1021/acsomega.5c04929 PMC12573176

[89] J. Hu, M. Li, Z. Liu, Y. Ding, Y. Sun et al., Tailoring the functionalities of MoS_2_ field-effect transistors by an area-selective surface charge transfer doping strategy. Nano Res. **18**(5), 94907360 (2025). 10.26599/nr.2025.94907360

[90] Z. Zhang, S. Huo, Q. Tian, F. Meng, Z. Yang et al., Near-perfect standard ternary inverter based on MoTe_2_ homojunction anti-ambipolar transistor. Adv. Funct. Mater. **35**(29), 2424728 (2025). 10.1002/adfm.202424728

[91] Q. Cui, H. Shou, C. Wu, B. Tang, W. Zhu et al., Growth of monolayer WS_2_ lateral homojunctions *via in situ* domain engineering. J. Am. Chem. Soc. **147**(25), 21778–21788 (2025). 10.1021/jacs.5c04546 40512549 10.1021/jacs.5c04546

[92] Y. Beckmann, A. Grundmann, L. Daniel, M. Abdelbaky, C. McAleese et al., Role of surface adsorbates on the photoresponse of (MO)CVD-grown graphene–MoS_2_ heterostructure photodetectors. ACS Appl. Mater. Interfaces **14**(30), 35184–35193 (2022). 10.1021/acsami.2c06047 35852455 10.1021/acsami.2c06047

[93] J. Oswald, D. Beretta, M. Stiefel, R. Furrer, D. Vuillaume et al., The effect of C60 and pentacene adsorbates on the electrical properties of CVD graphene on SiO_2_. Nanomaterials **13**(6), 1134 (2023). 10.3390/nano13061134 36986028 10.3390/nano13061134 PMC10052095

[94] S. Deng, H. Yu, T.J. Park, A.N.M.N. Islam, S. Manna et al., Selective area doping for Mott neuromorphic electronics. Sci. Adv. **9**(11), eade4838 (2023). 10.1126/sciadv.ade4838 36930716 10.1126/sciadv.ade4838 PMC10022892

[95] F. Wang, K. Pei, Y. Li, H. Li, T. Zhai, 2D homojunctions for electronics and optoelectronics. Adv. Mater. **33**(15), 2005303 (2021). 10.1002/adma.202005303 10.1002/adma.202005303 33644885

[96] S. Yang, G. Lee, J. Kim, S. Yang, C.-H. Lee, An in-plane WSe_2_ p–n homojunction two-dimensional diode by laser-induced doping. J. Mater. Chem. C **8**(25), 8393–8398 (2020). 10.1039/d0tc01790f

[97] S. Aftab, H.H. Hegazy, M.Z. Iqbal, M.W. Iqbal, G. Nazir et al., Recent advances in dynamic homojunction PIN diodes based on 2D materials. Adv. Mater. Interfaces **10**(6), 2201937 (2023). 10.1002/admi.202201937

[98] H. Park, J. Kim, Programmable synapse-like MoS_2_ field-effect transistors phase-engineered by dynamic lithium ion modulation. Adv. Electron. Mater. **6**(5), 1901410 (2020). 10.1002/aelm.201901410

[99] B. Zhao, Z. Xin, Y.-C. Wang, C. Wu, W. Wang et al., Bioinspired gas-receptor synergistic interaction for high-performance two-dimensional neuromorphic devices. Matter **8**(4), 102044 (2025). 10.1016/j.matt.2025.102044

[100] L. Liu, P. Gao, M. Zhang, J. Dou, C. Liu et al., Two-dimensional MoS_2_-based anisotropic synaptic transistor for neuromorphic computing by localized electron beam irradiation. Adv. Sci. **11**(45), 2408210 (2024). 10.1002/advs.202408210 10.1002/advs.202408210 PMC11615781 39413365

[101] K. Sun, J. Chen, X. Yan, The future of memristors: materials engineering and neural networks. Adv. Funct. Mater. **31**(8), 2006773 (2021). 10.1002/adfm.202006773

[102] P. Thakkar, J. Gosai, H.J. Gogoi, A. Solanki, From fundamentals to frontiers: a review of memristor mechanisms, modeling and emerging applications. J. Mater. Chem. C **12**(5), 1583–1608 (2024). 10.1039/D3TC03692H

[103] K.-S. Li, M.-K. Huang, Y.-H. Wang, Y.-C. Tseng, C.-J. Su, Wafer-scale fabrication of Al/MoS_2_/poly-Si memristors and insight of mechanism on the resistive switching. ACS Appl. Electron. Mater. **6**(2), 777–784 (2024). 10.1021/acsaelm.3c01314

[104] J. Zhou, X. Zhang, Y. Zhang, S. Huang, A. Chen et al., Hardware-implemented DropConnect function for energy-efficient neuromorphic computing. Adv. Funct. Mater. **35**(41), 2503452 (2025). 10.1002/adfm.202503452

[105] X. Wang, Q. He, H. Li, X. Zhang, H. Wang et al., Bio-inspired wide-field visual neuron implemented with ultra-low information loss population coding. Adv. Mater. **38**(2), e08803 (2026). 10.1002/adma.202508803 40984785 10.1002/adma.202508803

[106] M. Li, Z. Liu, Y. Sun, Y. Ding, H. Chen et al., Tailoring neuroplasticity in a ferroelectric-gated multi-terminal synaptic transistor by bi-directional modulation for improved pattern edge recognition. Adv. Funct. Mater. **33**(46), 2307986 (2023). 10.1002/adfm.202307986

[107] B.F. Yang, C. Zhang, Z.H. Zhang, D. Wang, Z.X. Wu et al., An InGaZnO synaptic transistor using titanium-oxide traps at back channel for neuromorphic computing. IEEE Trans. Electron Devices **72**(6), 2943–2948 (2025). 10.1109/TED.2025.3558719

[108] M. Li, Y. Ding, S. Zhao, H. Wang, Z. Liu et al., Spatial information inference with programmable 2D retinomorphic devices enabling dynamic trace perception inspired by bee waggle-dance communication. Adv. Funct. Mater. **36**(12), e16349 (2026). 10.1002/adfm.202516349

[109] S. Nam, D. Kang, S.-P. Jeon, D. Nam, J.-W. Jo et al., Contact-engineered oxide memtransistors for homeostasis-based high-linearity and precision neuromorphic computing. Small **21**(7), 2409510 (2025). 10.1002/smll.202409510 10.1002/smll.202409510 39757564

[110] W. Dong, S. Wang, B. Zhao, C. Xu, Y. Liu et al., Planar p–n junction engineering toward reconfigurable organic synaptic transistors for high-accuracy neuromorphic recognition. Small **21**(29), 2502740 (2025). 10.1002/smll.202502740 10.1002/smll.202502740 40401401

[111] Y. Zhou, L. Tong, Z. Chen, L. Tao, Y. Pang et al., Contact-engineered reconfigurable two-dimensional Schottky junction field-effect transistor with low leakage currents. Nat. Commun. **14**, 4270 (2023). 10.1038/s41467-023-39705-w 37460531 10.1038/s41467-023-39705-w PMC10352327

[112] X. Zhang, D. Qiu, P. Hou, Plasmonic hot-electron effect enhanced WSe_2_ based transistor based on asymmetric Schottky contacts for self-powered photodetection and visual synapse. ACS Appl. Mater. Interfaces **17**(21), 31543–31552 (2025). 10.1021/acsami.5c01347 40373283 10.1021/acsami.5c01347

[113] P. Wang, T. Zhao, S. Xiao, Q. Chen, Y. Zhang et al., Geometry-derived asymmetric Schottky contacts based on chemical vapor deposited MoS_2_. ACS Appl. Electron. Mater. **6**(10), 7475–7483 (2024). 10.1021/acsaelm.4c01338

[114] Z. Cheng, J. Backman, H. Zhang, H. Abuzaid, G. Li et al., Distinct contact scaling effects in MoS_2_ transistors revealed with asymmetrical contact measurements. Adv. Mater. **35**(21), 2210916 (2023). 10.1002/adma.202210916 10.1002/adma.202210916 36848627

[115] C. Liu, T. Zheng, K. Shu, S. Shu, Z. Lan et al., Polarization-sensitive self-powered Schottky photodetector with high photovoltaic performance induced by geometry-asymmetric contacts. ACS Appl. Mater. Interfaces **16**(11), 13914–13926 (2024). 10.1021/acsami.3c16047 38447591 10.1021/acsami.3c16047

[116] K. Tang, C. Yan, X. Du, G. Rao, M. Zhang et al., Asymmetric contacts on narrow-bandgap black phosphorus for self-driven broadband photodetectors. Adv. Opt. Mater. **12**(2), 2301350 (2024). 10.1002/adom.202301350

[117] J. Liu, K. Xing, L. Li, W. Zhao, A. Stacey et al., One-step transfer of symmetric and asymmetric contacts for large-scale 2D electronics and optoelectronics. ACS Nano **19**(30), 27919–27929 (2025). 10.1021/acsnano.5c09815 40698618 10.1021/acsnano.5c09815 PMC12333430

[118] H. Song, Y. Li, S. Liu, X. Zhou, Y. Zhou et al., Reconfigurable graded adaptive asymmetry-Schottky-barrier phototransistor for artificial visual system with zJ-energy record. Appl. Phys. Rev. **12**(2), 021418 (2025). 10.1063/5.0257883

[119] H. Wu, Y. Cui, J. Xu, Z. Yan, Z. Xie et al., Multifunctional half-floating-gate field-effect transistor based on MoS_2_–BN–graphene van der Waals heterostructures. Nano Lett. **22**(6), 2328–2333 (2022). 10.1021/acs.nanolett.1c04737 35254079 10.1021/acs.nanolett.1c04737

[120] Y. Song, Z. Pan, C. Luo, Y. Wang, T. Zheng et al., Ferroelectric α-In_2_Se_3_ semi-floating gate transistors for multilevel memory and optoelectronic logic gate. ACS Appl. Mater. Interfaces **17**(18), 26901–26907 (2025). 10.1021/acsami.5c01586 40267295 10.1021/acsami.5c01586

[121] Y. Pang, Y. Zhou, L. Tong, J. Xu, 2D dual gate field-effect transistor enabled versatile functions. Small **20**(2), 2304173 (2024). 10.1002/smll.202304173 10.1002/smll.202304173 37705128

[122] Y. Zhu, Y. Wang, X. Pang, Y. Jiang, X. Liu et al., Non-volatile 2D MoS_2_/black phosphorus heterojunction photodiodes in the near- to mid-infrared region. Nat. Commun. **15**, 6015 (2024). 10.1038/s41467-024-50353-6 39019876 10.1038/s41467-024-50353-6 PMC11255212

[123] S. Zhang, S. Zhu, S. Tian, L. Zhang, C. Chen et al., Polarization-sensitive neuromorphic vision sensing enabled by pristine black arsenic-phosphorus. Light Sci. Appl. **15**, 100 (2026). 10.1038/s41377-025-02125-0 41629281 10.1038/s41377-025-02125-0 PMC12865036

[124] A. Rehmat, M. Asim, M. Hamza Pervez, M. Asghar Khan, S.H. Shin et al., Floating gate synaptic memory of Janus WSSe Multilayer for neuromorphic computing. Mater. Today Adv. **27**, 100608 (2025). 10.1016/j.mtadv.2025.100608

[125] X. Wen, Z. Wang, T. Wang, J. Meng, Brain-inspired two-dimensional CuInP_2_S_6_ ferroelectric materials for neuromorphic computing. Adv. Funct. Mater. (2026). 10.1002/adfm.202530852

[126] Z. Lian, J. Wei, Y. Liu, Z. Liu, Y. Liu et al., Highly responsive dual-function deep-ultraviolet neuromorphic phototransistors based on silicon carbide nanoparticle/2D MoS_2_ heterostructures. ACS Nano **19**(28), 26041–26054 (2025). 10.1021/acsnano.5c06692 40644499 10.1021/acsnano.5c06692

[127] Z. Lin, J. Chen, Z. Zheng, Q. Lai, Z. Liu et al., Multifunctional UV photodetect-memristors based on area selective fabricated Ga_2_S_3_/graphene/GaN van der Waals heterojunctions. Mater. Horiz. **12**(9), 3091–3104 (2025). 10.1039/D4MH01711K 39878536 10.1039/d4mh01711k

[128] S. Ji, J. Kim, J. Hong, J. Choi, S. Yun et al., 2D material-based memristor arrays for flexible and thermally stable neuromorphic applications. Small (2025). 10.1002/smll.202507845 10.1002/smll.202507845 41307195

[129] X. Zhang, M. Chi, S. Tian, J. Zhang, T. Xu et al., Asymmetric ferroelectric gated reconfigurable WSe_2_ p–n homojunction for in-sensor neuromorphic vision processing. Adv. Funct. Mater. **36**(17), e20019 (2026). 10.1002/adfm.202520019

[130] C. Mahata, D. Ju, T. Das, B. Jeon, M. Ismail et al., Artificial synapses based on 2D-layered palladium diselenide heterostructure dynamic memristor for neuromorphic applications. Nano Energy **120**, 109168 (2024). 10.1016/j.nanoen.2023.109168

[131] S. Pazos, K. Zhu, M.A. Villena, O. Alharbi, W. Zheng et al., Synaptic and neural behaviours in a standard silicon transistor. Nature **640**(8057), 69–76 (2025). 10.1038/s41586-025-08742-4 40140586 10.1038/s41586-025-08742-4 PMC11964925

[132] J. Park, A. Kumar, Y. Zhou, S. Oh, J.-H. Kim et al., Multi-level, forming and filament free, bulk switching trilayer RRAM for neuromorphic computing at the edge. Nat. Commun. **15**, 3492 (2024). 10.1038/s41467-024-46682-1 38664381 10.1038/s41467-024-46682-1 PMC11045755

[133] S.-Y. Kang, S.-M. Jin, J.-Y. Lee, D.-S. Woo, T.-H. Shim et al., Layer-dependent effects of interfacial phase-change memory for an artificial synapse. physica status solidi (RRL) – Rapid Research Letters **16**(9), 2100616 (2022). 10.1002/pssr.202100616

[134] V.K. Sangwan, H.-S. Lee, H. Bergeron, I. Balla, M.E. Beck et al., Multi-terminal memtransistors from polycrystalline monolayer molybdenum disulfide. Nature **554**(7693), 500–504 (2018). 10.1038/nature25747 29469093 10.1038/nature25747

[135] W. Huh, D. Lee, S. Jang, J.H. Kang, T.H. Yoon et al., Heterosynaptic MoS_2_ memtransistors emulating biological neuromodulation for energy-efficient neuromorphic electronics. Adv. Mater. **35**(24), 2211525 (2023). 10.1002/adma.202211525 10.1002/adma.202211525 36930856

[136] X. Wang, B. Wang, Q. Zhang, Y. Sun, E. Wang et al., Grain-boundary engineering of monolayer MoS_2_ for energy-efficient lateral synaptic devices. Adv. Mater. **33**(32), 2102435 (2021). 10.1002/adma.202102435 10.1002/adma.202102435 34219298

[137] M. Lanza, A. Sebastian, W.D. Lu, M.L. Gallo, M.-F. Chang et al., Memristive technologies for data storage, computation, encryption, and radio-frequency communication. Science **376**(6597), eabj9979 (2022). 10.1126/science.abj9979 35653464 10.1126/science.abj9979

[138] J. Yang, A. Yoon, D. Lee, S. Song, I.J. Jung et al., Wafer-scale memristor array based on aligned grain boundaries of 2D molybdenum ditelluride for application to artificial synapses. Adv. Funct. Mater. **34**(15), 2309455 (2024). 10.1002/adfm.202309455

[139] R.R. Das, T.R. Rajalekshmi, S. Pallathuvalappil, A. James, FETs for analog neural MACs. IEEE Access **12**, 54019–54048 (2024). 10.1109/ACCESS.2024.3387094

[140] K. Zhu, S. Pazos, F. Aguirre, Y. Shen, Y. Yuan et al., Hybrid 2D–CMOS microchips for memristive applications. Nature **618**(7963), 57–62 (2023). 10.1038/s41586-023-05973-1 36972685 10.1038/s41586-023-05973-1 PMC10232361

[141] R. Zhao, T. Wang, T. Moon, Y. Xu, J. Zhao et al., A spiking artificial neuron based on one diffusive memristor, one transistor and one resistor. Nat. Electron. **8**(12), 1211–1221 (2025). 10.1038/s41928-025-01488-x

[142] W. Wang, Y. Li, M. Wang, Difficulties and approaches in enabling learning-in-memory using crossbar arrays of memristors. Neuromorph. Comput. Eng. **4**(3), 032002 (2024). 10.1088/2634-4386/ad6732

[143] T. Nazeer, S.A. Ahsan, Physics-based SPICE model of 2D-material FETs for neuromorphic circuit simulation. 2025 International Compact Modeling Conference (ICMC)., 1–4. IEEE (2025). 10.1109/ICMC64879.2025.11102634

[144] Y. Shi, N.T. Duong, K.-W. Ang, Emerging 2D materials hardware for in-sensor computing. Nanoscale Horiz. **10**(2), 205–229 (2025). 10.1039/d4nh00405a 39555812 10.1039/d4nh00405a

[145] G. Zhang, Q. Luo, J. Yao, S. Zhong, H. Wang et al., All-in-one neuromorphic hardware with 2D material technology: current status and future perspective. Chem. Soc. Rev. **54**(18), 8196–8242 (2025). 10.1039/D5CS00251F 40761130 10.1039/d5cs00251f

[146] J.-H. Kang, H. Shin, K.S. Kim, M.-K. Song, D. Lee et al., Monolithic 3D integration of 2D materials-based electronics towards ultimate edge computing solutions. Nat. Mater. **22**(12), 1470–1477 (2023). 10.1038/s41563-023-01704-z 38012388 10.1038/s41563-023-01704-z

[147] J. Zhang, C. Shang, X. Dai, Y. Zhang, T. Zhu et al., Effective passivation of anisotropic 2D GeAs *via* graphene encapsulation for highly stable near-infrared photodetectors. ACS Appl. Mater. Interfaces **15**(10), 13281–13289 (2023). 10.1021/acsami.2c20030 36857585 10.1021/acsami.2c20030

[148] S.J. Kim, H.-J. Lee, C.-H. Lee, H.W. Jang, 2D materials-based 3D integration for neuromorphic hardware. npj 2D Mater. Appl. **8**, 70 (2024). 10.1038/s41699-024-00509-1

